# Design, synthesis and biological activity of glycoconjugated ADAMTS5 exosite inhibitors: applications in osteoarthritis and ovarian cancer models

**DOI:** 10.1038/s41598-025-29549-3

**Published:** 2025-12-11

**Authors:** Doretta Cuffaro, Sophie Blagg, Kazuhiro Yamamoto, Luca Pinzi, Rachele Bacchetti, Shengnan Yuan, Simon Tew, Paola Campagnolo, Felicia D’Andrea, Enrico Crispino, Giulio Rastelli, Armando Rossello, Elena Rainero, Elisa Nuti, Salvatore Santamaria

**Affiliations:** 1 Department of Pharmacy, University of Pisa, Via Bonanno 6, Pisa, 56126 Italy; 2 Department of Immunology and Inflammation, Imperial College London, Du Cane Road, London, W12 0NN UK; 3 Institute of Life Course and Medical Sciences, University of Liverpool, Liverpool, L7 8TX UK; 4 Scleroprotein and Leather Research Institute, Faculty of Agriculture, Tokyo University of Agriculture and Technology, 3-5-8 Saiwaicho, Fuchu, 183-8509 Tokyo Japan; 5 Department of Life Sciences, University of Modena and Reggio Emilia, Via Giuseppe Campi, 103, Modena, 41125 Italy; 6 School of Biosciences, University of Sheffield, Western Bank, Sheffield, S10 2TN UK; 7 Discipline of Clinical Sciences, School of Biosciences, University of Surrey, Guildford, GU2 7XH Surrey UK; 8 Department of Comparative Biomedical Sciences, School of Veterinary Medicine, Guildford, GU2 7XH Surrey UK

**Keywords:** ADAMTS5, Aggrecan, Versican, Glycoconjugates, Osteoarthritis, Ovarian cancer, Biochemistry, Cell biology, Drug discovery

## Abstract

Pharmacological inhibition of the extracellular zinc metalloprotease A Disintegrin-like And Metalloprotease domain with Thrombospondin type I motifs 5 (ADAMTS5) has been proposed as a treatment for osteoarthritis (OA), a degenerative disease characterized by cartilage loss. More recently, ADAMTS5 has been implicated in ovarian cancer (OC), due to its essential role in promoting cell migration and association with poor prognosis. ADAMTS5 major substrates are the proteoglycans aggrecan and versican, which support the structural integrity of the cartilage and the tumor microenvironment. We have recently described a non-chelating arylsulfonamide glycoconjugate, compound **4b**, as a selective ADAMTS5 inhibitor, and shown its effectiveness in an OC 3D model. Here, we modified the structure of **4b** to improve its biological activity. We showed that, while **4b** induces cytotoxicity in several cell lines as well as in porcine and human cartilage explants, its derivative **2** was tolerated at high micromolar concentrations and effective in inhibiting aggrecan degradation in human *ex vivo* OA explants and reducing directional OC cell migration and pseudopod elongation. *In silico* analyses provided a rationale behind the different biological activities of the two compounds. These findings highlight the potential of non-chelating glycoconjugated arylsulfonamides to treat pathologies characterized by excessive ADAMTS5 activity.

**Supplementary Information:**

The online version contains supplementary material available at 10.1038/s41598-025-29549-3.

## Introduction

A Disintegrin-like And Metalloprotease domain with Thrombospondin type I motifs (ADAMTS) 5 is a secreted zinc metalloprotease playing a crucial role in regulating extracellular levels of the large aggregating proteoglycans aggrecan and versican^1^. Proteoglycans exert a Gibb-Donnan effect through the ion-binding ability of their sulfated glycosaminoglycan (GAG) chains and are therefore responsible for the viscoelastic properties of most connective tissues^2^. Consequently, dysregulated ADAMTS5 activity compromises the structural integrity of the extracellular matrix, contributing to several pathologies such as osteoarthritis (OA) and cancer^1,3,4^.

OA is the most common chronic joint disease and a leading cause of pain and disability in developed countries, the lifetime risk of developing symptomatic knee OA being around 40%^5^. It is estimated that by 2050 OA will affect 130 million people worldwide^6^. Currently, OA treatment is limited to steroidal and non-steroidal anti-inflammatory drugs that reduce pain and inflammation but do not arrest or slow down the progression of the disease^7^. Joint replacement surgery remains the only option to restore mobility. Development of disease-modifying OA drugs will improve life of OA patients and arrest cartilage degradation, thus reducing the necessity for surgery. Excessive proteolysis of aggrecan, the major structural proteoglycan in articular cartilage, represents an early, reversible event in the development of OA.

Research in the past 25 years has identified ADAMTS5 (aggrecanase-2), as a valid therapeutic target for OA^1^. ADAMTS5 is 20/30-fold more potent aggrecanase than ADAMTS4 (aggrecanase-1), a closely related ADAMTS family member^8^. In contrast to *Adamts4* knockout mice^9^, *Adamts5* knockout mice showed protection from cartilage degradation in experimental OA models^10,11^. Moreover, anti-ADAMTS5 inhibitory monoclonal antibodies are effective in blocking aggrecan degradation both *ex vivo* and *in vivo*^12–16^.

Recently, we have shown that ADAMTS5 expression is necessary and sufficient to stimulate migration of ovarian cancer (OC) cells and that is correlated with poor prognosis in OC patients^17^. In OC, ADAMTS5 pathological action is most likely mediated by proteolysis of versican rather than aggrecan. Versican is an anti-adhesive proteoglycan known to stimulate OC cell migration and invasion as well as limit the ability of T cells to infiltrate the tumor^18,19^. Versican anti-adhesive properties are enhanced when it is cleaved by ADAMTS5 and other ADAMTS versicanases at E^441^-A^442^ (V1 isoform numbering, UniProt ID P13611-2), thus releasing an N-terminal bioactive fragment named versikine^20,21^. ADAMTS5 inhibitors may therefore find applications in OA, OC, and potentially, other carcinomas (reviewed in^22^.

**Fig. 1:**
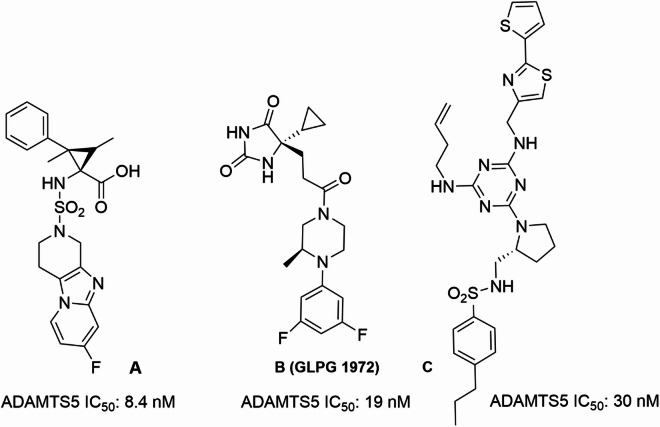
Structures of reported selective zinc-chelating ADAMTS5 inhibitors and their corresponding inhibitory activities.

However, development of selective ADAMTS5 inhibitors has been hampered by the highly conserved geometry of the active site in the ADAMTS family (19 members in humans) as well as in other closely related families such as the Disintegrin-like And Metalloproteases (21 members), and Matrix Metalloproteases (23 members), all belonging to the metzincin superfamily^23^. The high structural conservation in metzincins is thought to be at least partially responsible for the failure of broad-spectrum zinc-chelating matrix metalloprotease inhibitors in early clinical trials for anti-cancer therapy as cross-inhibition of anti-target metalloproteases caused unacceptable side effects such as musculoskeletal syndrome^24^. A few potent and selective zinc-chelating ADAMTS5 inhibitors have been recently reported (compounds **A-C**, Fig. 1)^25–27^, but none have yet reached the market. A way to address the selectivity issues is targeting non-conserved substrate-binding sites in the ancillary domains of ADAMTS5, i.e. exosites^28^, which have been identified in the disintegrin-like (Dis)^29^ and spacer domains^30^.

We have previously shown that a monoclonal antibody against the spacer domain acts as an exosite inhibitor, as it blocked ADAMTS5 aggrecanase and versicanase activities without affecting cleavage of peptide substrates engaging the active site and its surroundings^12,30^. This discovery opened the possibility to use exosite inhibitors to target specifically ADAMTS5 proteoglycanase activity. From a translational perspective, however, antibodies have some limitations, in particular for OA treatment, given the need for intra-articular injections for extended periods of time, a burden for both patients and medical practitioners^31^.

**Fig. 2:**
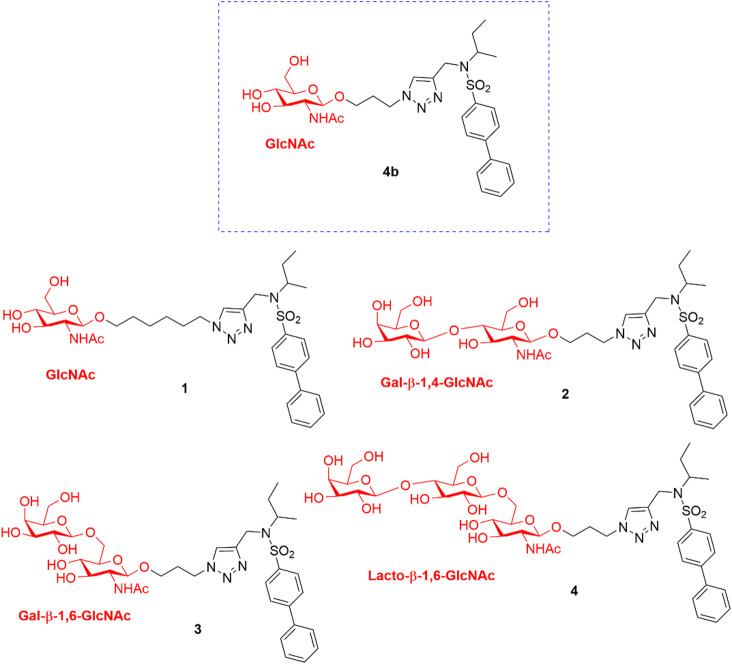
Analogues of compound 4b synthesized in the present study. The carbohydrate moiety is highlighted in red.

**Fig. 3:**
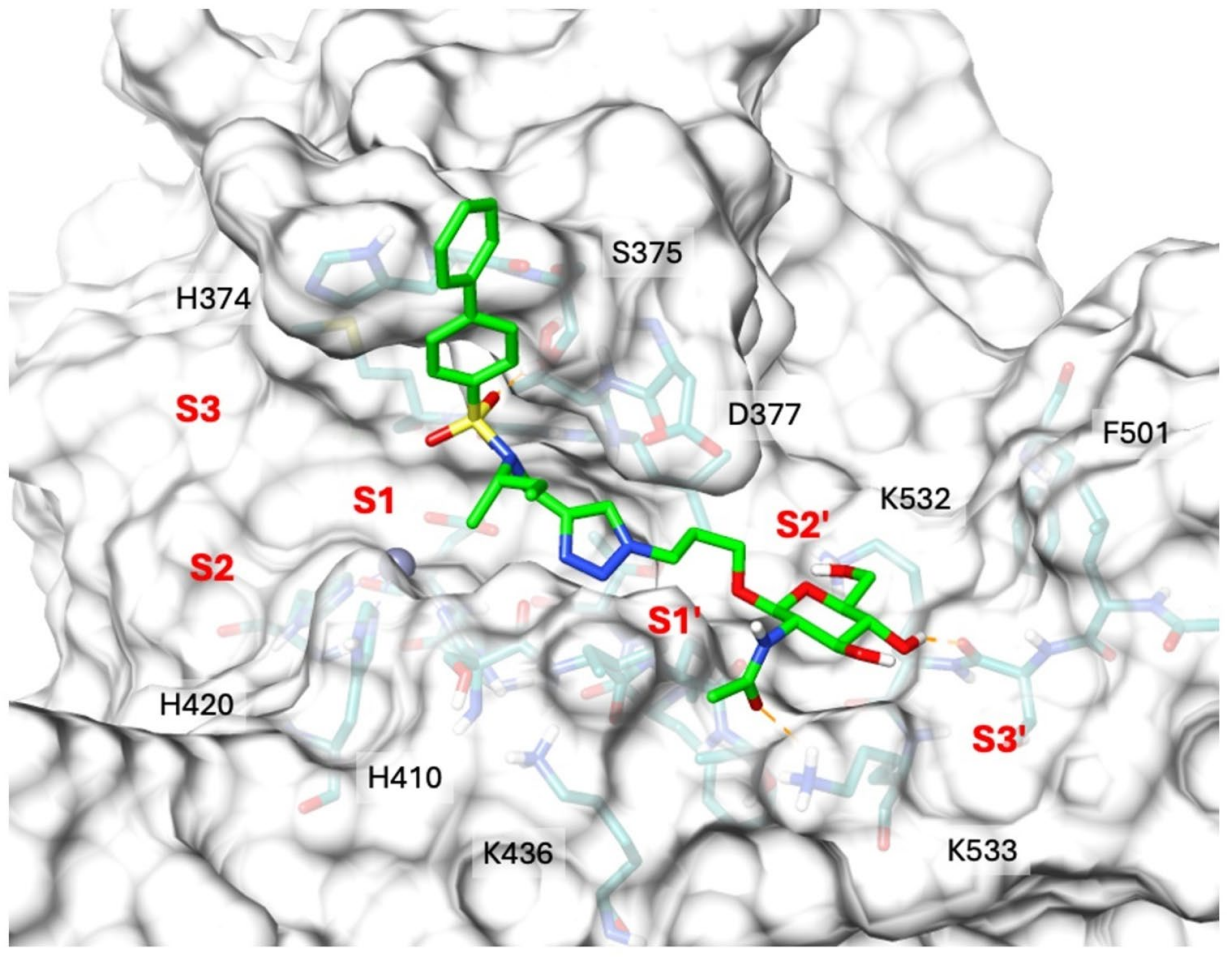
Binding interactions of 4b with ADAMTS5 metalloproteinase/Dis domains. H-bond interactions are shown as light orange dashed lines. K532 and K533 constitute an exosite in the Dis domain.

An alternative to antibodies is represented by GAGs and their derivatives. GAGs are long linear polysaccharides consisting of repeating disaccharide units covalently attached to serine residues in the protein core of proteoglycans. Heparin, a GAG whose most common disaccharide unit is composed of a 2-*O*-sulfated iduronic acid and 6-*O*-sulfated, *N*-sulfated β-*N*-acetyl-d-glucosamine (GlcNAc), inhibits ADAMTS5 proteoglycanase activity by binding to exosites located in the spacer and cysteine-rich domains, albeit at high micromolar concentrations^8,32,33^. However, due to its low affinity, as well as anticoagulant properties and associated side effects^34,35^, heparin itself is not a suitable disease-modifying OA agent. To identify glycoconjugates with improved inhibitory activity, we probed ADAMTS5 with a small library of molecules containing the GlcNAc moiety and lacking a zinc-binding group (ZBG). This led to the identification of the glycoconjugated arylsulfonamide **4b**, which inhibited ADAMTS5 activity against versican and aggrecan while sparing its peptidolytic activity^29^ **(**Fig. 2**)**. **4b** is an ADAMTS5 exosite inhibitor which does not interact with the active site zinc but instead binds through two contiguous positively charged residues (^532^KK^533^, UniProt ID Q9UNA0) in the Dis domain through its GlcNAc moiety (Fig. 3). Importantly, **4b** does not inhibit ADAMTS4, a proteoglycanase closely related to ADAMTS5, thus showing selectivity^29^. Recently, we have shown that **4b** is also effective in reducing OC spheroid invasion^17^. These findings strongly suggest that GAG derivatives can represent novel exosite inhibitors of ADAMTS5. Here, we aim to modify the structure of compound **4b** in order to improve its biological activity in an *ex vivo* OA model and OC 3D systems.

## Results and discussion

### Design strategy

Using molecular dynamics simulations combined with docking calculations and experimental validation by in vitro enzymatic assays, we have previously demonstrated that the GlcNAc moiety of compound **4b** showed H-bond interactions with residues ^532^KK^533^ in the Dis domain of ADAMTS5, approximately 17–18 Å from the catalytic zinc ion (Fig. 3)^29^. The biphenyl group of the arylsulfonamide occupies the S1’ pocket but does not bind to the active site zinc, as shown by its ability to bind in the presence of the zinc-chelator GM6001 (Ilomastat)^29^.

We aimed to preserve the GlcNAc monosaccharide unit responsible for interaction with residues ^532^KK^533^ in the ADAMTS5 Dis domain, explore other glycosidic units, i.e., either glucose (Glu) or galactose (Gal), in 6 or in 4-position, or modify the length of the alkoxy linker between the sugar moiety and the arylsulfonamide (Fig. 2). The length of the linker could be crucial for the proper positioning of the inhibitor when binding to ADAMTS5 metalloproteinase and Dis domains. A longer linker might allow better interaction with the enzyme by increasing the flexibility of the molecule, thereby facilitating the correct placement of the biphenyl group within the S1’ pocket while maintaining the interaction between the GlcNAc unit and residues ^532^KK^533^ (compound **1**). Alternatively, we hypothesized that additional monosaccharide units (compounds **2–4**) could establish stronger interactions with ^532^KK^533^ and/or additional interactions with other residues in the metalloproteinase domain to improve biological activity.

### Chemistry

**Scheme 1:**
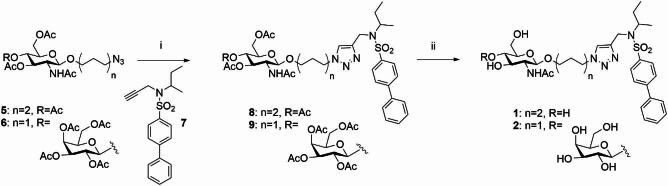
Reagents and conditions: (i) **7**, sodium ascorbate, CuSO_4_, DMF/H_2_O 4:1, MW, 100 °C, 45 min; (ii) NH_3_-MeOH 7 N, MeOH, r t, 3.5 h, (**1** = over two steps 82%; **2** = over two steps 42%).

The synthesis of compounds **1** and **2** is reported in Scheme 1.

Starting from the known azido derivatives **5**^36^ or **6**^37^ and the alkynyl sulfonamide **7**^29^, the glycoconjugates **8** and **9** were synthesized via a click chemistry reaction, incorporating a 1,2,3-triazole linker, by a Cu(I)-catalyzed Huisgen 1,3-dipolar cycloaddition (CuAAC). Specifically, the reaction was conducted using microwave irradiation at 100 °C in a mixture containing the appropriate azido derivative, alkyne **7**, copper sulfate (CuSO₄), sodium ascorbate, and DMF/H₂O mixture (4:1 ratio). In both cases, the resulting glycoconjugate, **8** or **9**, was directly subjected, without further purification, to a basic hydrolysis with methanolic ammonia (NH₃-MeOH, 7 N) to deprotect the hydroxyl groups of the sugar moiety affording the desired glycoconjugate **1** or **2** (**1** overall yield from **5** in two steps: 82%; **2** overall yield from **6** in two steps: 42%).

**Scheme 2:**
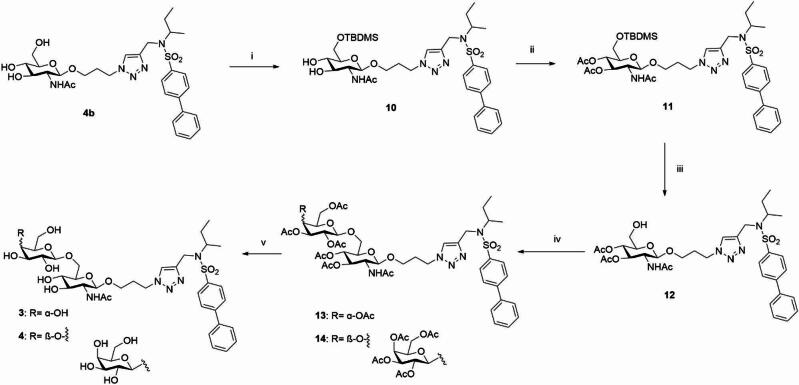
Reagents and conditions: (i) TBDMSCl, Py, 0 °C, 1 h (78%); (ii) Ac_2_O, Py, rt, 16 h, (94%); (iii) AcOH aq 70%, 70 °C, 1.5 h, (87%); (iv) 2,3,4,6-tetra-*O*-acetyl-α-d-galactopyranosyl bromide, AgOTf, DCM dry, − 15 °C to rt, 26 h; or peracetyl-lactose trichloroacetoimidate, BF_3_ Et_2_O, dry DCM, − 15 °C to rt, 12 h; (v) NH_3_-MeOH 7 N, MeOH, rt, 5–21 h (**3**: over two steps from **12**: 15%; **4**: over two steps from **12**: 40%).

For the synthesis of the final compounds **3** and **4**, containing respectively a disaccharide and trisaccharide unit with a β-1,6-glycosidic bond, it was necessary to employ various orthogonal protecting groups to selectively deprotect the 6-position of the GlcNAc involved in the subsequent glycosylation reaction **(**Scheme 2**)**. Considering the structure of compound **4b**, the hydroxyl group at position 6, as a primary alcohol, is more reactive than the secondary hydroxyl groups at C-3 and C-4 due to its higher nucleophilicity. This allowed to regioselectively protect the **4b**-C-6 hydroxyl group under controlled conditions with a bulky protecting group such as *tert*-butyldimethylsilyl (TBDMS). The hydroxyl groups at C-3 and C-4, exhibiting comparable reactivity, were protected by acetylation, taking advantage of the orthogonality between acetyl and silyl protecting groups.

Starting from compound **4b**, compound **10** was synthesized by selectively protecting the hydroxyl group at C-6 using *tert*-butyldimethylsilyl chloride (TBDMSCl) in anhydrous pyridine. Subsequently, the hydroxyl groups at C-3 and C-4 of compound **10** were protected as acetates through classical acetylation reaction with acetic anhydride in anhydrous pyridine, yielding compound **11**. The silyl derivative **11** was then subjected to selective deprotection of the C-6 hydroxyl group by acidic hydrolysis (70% aqueous AcOH) at 70 °C, resulting in compound **12**. Compound **12** was used as the glycosyl acceptor in two different glycosylation reactions (β-galactosylation and β-lactosylation) with the commercially available glycosyl donors 2,3,4,6-tetra-*O*-acetyl-α- d-galactopyranosyl bromide or hepta-*O*-acetyl-1-α-*O*-lactose trichloroacetimidate, respectively. The glycosylation reactions were conducted under anhydrous conditions, silver triflate (AgOTf) or BF_3_ Et_2_O were added as catalyst activators at -15 °C, and the temperature was then gradually increased to ambient temperature to promote the reaction. The stereoselective formation of the new β-glycosidic bonds are generally facilitated by the presence of an acyl group (in this case, an ester group) at the C-2 position of the glycosyl donor. Specifically, the departure of the leaving group from the anomeric carbon is anchimerically assisted by the carbonyl function, leading to the formation of a 1,2-acyloxonium ion, which is a thermodynamically stable cyclic carbocation. The subsequent nucleophilic attack on the anomeric carbon occurs from the less sterically hindered side of the acyloxonium ion, resulting in the formation of the β-glycoside and the regeneration of the acyl group at C-2. The reaction resulted in the formation of the desired disaccharide **13** or trisaccharide **14**. Starting from compounds **13** and **14**, all hydroxyl groups were deprotected via basic hydrolysis with NH_3_-MeOH 7 N, in methanol, yielding the final disaccharide **3** (overall yield over two steps from compound **12**: 15%) and trisaccharide **4** (overall yield over two steps from compound **12**: 40%).

### *In vitro* inhibitory activity of **4b** derivatives

The clear-cut test to ascertain if an inhibitor binds an exosite rather than the active site is comparing the inhibitory activity of the molecule against two different substrates, a quenched-fluorescent (QF) peptide and a protein substrate^12,28,29^. While a short QF peptide engages residues surrounding the active site, proteolysis of native substrates involves both the active site and exosites. Therefore, a truly exosite inhibitor of ADAMTS5 will inhibit exclusively cleavage of proteoglycans^12,28,29^. To test if the derivatives of compound **4b** act as exosite inhibitors, their inhibitory activity against ADAMTS5 and closely related ADAMTS4 was tested using both a QF peptide cleavage assay and a quantitative versicanase assay^30,38^. Importantly, we demonstrated that ADAMTS5 employs the same exosites to recognize both aggrecan and versican^29,30^.

**Fig. 4:**
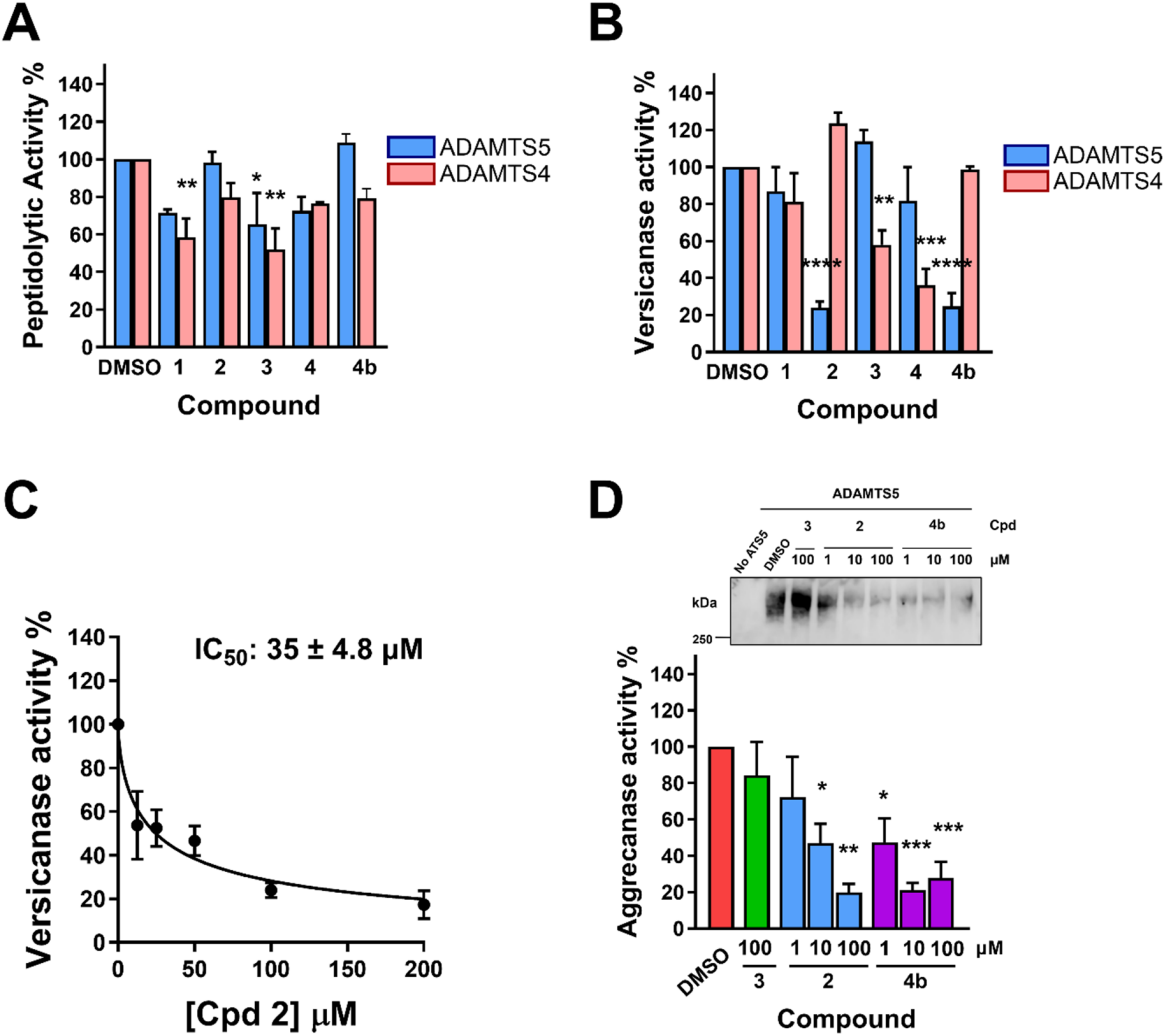
Inhibitory activity of compounds 1–4 against ADAMTS4 and ADAMTS5. (**A**) Inhibition of ADAMTS4 and ADAMTS5 peptidolytic activity . (**B**) Inhibition of ADAMTS4 and ADAMTS5 versicanase activity. (**C**) Dose-response curve for inhibition of ADAMTS5 versicanase activity by compound 2. (**D**) Inhibition of ADAMTS5 aggrecanase activity. Compounds were incubated with ADAMTS5 (1 nM) for 2 h at 37 °C before addition of aggrecan (600 nM). Following SDS-PAGE and immunoblot, fragments were detected by an aggrecan neoepitope antibody that recognizes the new C-terminal fragment generated by ADAMTS5 cleavage at E^392^-A^393^ and analyzed by densitometric analysis. A representative immunoblot is shown on the top of the panel. Data are presented as mean ± SEM (*n* = 3–4 independent experiments). Cpd, compound. *p < 0.05; **p < 0.005; ***p < 0.001; ****p < 0.0001 compared to DMSO control by one-way ANOVA.

With the exception of compound **3**, none of the compounds exhibited significant inhibition on the peptidolytic activity of ADAMTS5 at 100 µM compared to an equal concentration (v/v) of DMSO (hereinafter indicated as DMSO control), similarly to the parent compound, **4b (**Fig. 4A**)**. Compounds **1** and **3** significantly inhibited ADAMTS4 peptidolytic activity by 42 and 48%, respectively, suggesting that they bind closer to the active site than **4b (**Fig. 4A**)**. Only compound **2** significantly inhibited ADAMTS5 versicanase activity by 76% while sparing ADAMTS4, similar to the parent compound **4b** (Fig. 4B). Compounds **3** and **4** significantly inhibited versicanase ADAMTS4 activity by 42 and 64%, respectively. These results suggest that selectivity for ADAMTS5 depends on the presence of unbranched monosaccharides (as in compounds **1** and **2**). The IC_50_ value for inhibition of ADAMTS5 versicanase activity by compound **2** was 35 ± 4.8 µM **(**Fig. 4C**)**, ~ 4-fold higher than the parent compound **4b** (9.4 ± 2.8 µM^29^**)**, indicating that the addition of an unbranched monosaccharide decreases inhibitory activity against ADAMTS5. Compound **2** was then tested in an aggrecanase assay^39^ alongside compound **3** and **4b**, used as negative and positive controls, respectively. While compound **3** was ineffective at inhibiting ADAMTS5 aggrecanase activity at 100 µM, compound **2** significantly inhibited ADAMTS5 at 10 and 100 µM by 53 and 80%, respectively, slightly lower than **4b**, which showed significant inhibition at 1 (53%), 10 (79%) and 100 (72%) µM (Fig. 4D). These results confirmed the decreased inhibitory potency of compound **2** compared to **4b**. Nevertheless, compound **2** was selected for further characterization alongside the parent compound, **4b**, as it retained selective inhibitory activity against ADAMTS5, as well as an exosite-targeting mechanism.

### *In vitro *solubility of compounds **2** and **4b**

Before performing cell studies, the water solubility of compounds **2** and **4b** was determined. UV-Vis spectrophotometric analysis in phosphate buffered saline (PBS, pH 7.4) showed a linear correlation between absorbance at 284 nm and compound concentration in the range of 12.5–300 µM (**2**: y = 0.0063x + 0.1237, r^2^: 0.992; **4b**: y = 0.0064x + 0.0995, r^2^: 0.993) (**Supplementary Information Fig. ****S1**). No significant deviation was observed within this range, indicating that both compounds are freely soluble up to 300 µM under the tested conditions. Above 300 µM, a plateau in absorbance was observed for both compounds, indicating 300 µM as the upper solubility limit under the tested conditions.

### Cytotoxic effect of compounds **2** and **4b**

**Fig. 5:**
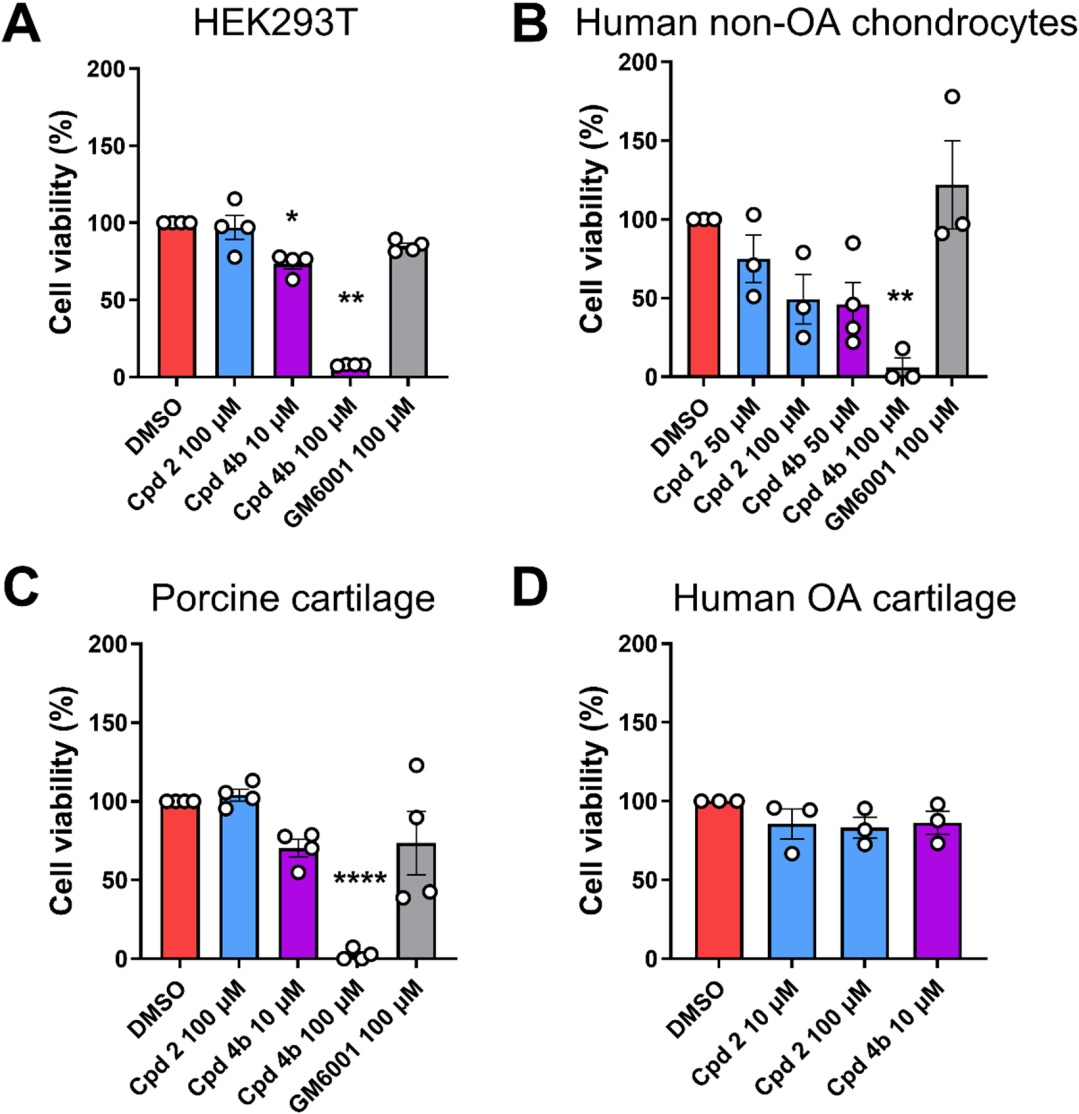
Effect of compounds 2 and 4b on cell viability. Compounds **2** and **4b** or DMSO control were incubated with HEK293T cells (**A**), human non-OA chondrocytes (**B**), porcine cartilage explants (**C**), or human OA cartilage explants (**D**) for 72 h. Cell viability was measured using MTS assay and reported as percentage of DMSO control. Data are plotted as scatter dot plots with average ± S.E.M. (*n* = 3–4 independent experiments with 3 technical replicates per experiment). *p < 0.05; **p < 0.005; ****p < 0.0001 compared to DMSO control by one-way ANOVA.

Before testing the biological activity of compounds **2** and **4b** in OA and OC models, we assessed their cytotoxic effects on human embryonic kidney cells expressing the SV40 large T antigen (HEK293T) using an assay based on the MTS (3-(4,5-dimethylthiazol-2-yl)-5-(3-carboxymethoxyphenyl)-2-(4-sulfophenyl)-2 H-tetrazolium) dye (Fig. 5A). At 10 and 100 µM, **4b** significantly inhibited cell viability by 22 and 92%, respectively, compared to the DMSO control. Neither **2**, nor the broad-spectrum zinc-chelating metalloproteinase inhibitor GM6001^40^ had an effect on cell viability at 100 µM. These findings were replicated in human chondrocytes isolated from non-OA patients, where **4b** significantly inhibited cell viability by 94% at 100 µM (Fig. 5B). At 50 µM, **4b** inhibited cell viability of human chondrocytes by 54%, although this effect was not significant. No significant effect was observed in the presence of compound **2** (50 and 100 µM) or GM6001 (100 µM). We hypothesized that as both HEK293T cells and human chondrocytes were grown in monolayers, which have a less dense and protective extracellular matrix, they were more susceptible to cytotoxic effects than chondrocytes embedded in their native cartilage. Therefore, compounds **2** and **4b** were tested on freshly isolated pig articular cartilage explants. Similar to monolayer cultures, the addition of **4b** at 100 µM decreased cell viability by ~ 97%, while no effect was observed at 10 µM (Fig. 5C). Compound **2** and GM6001 did not exhibit cytotoxicity at 100 µM. Based on these results, we decided to test the anti-aggrecanolytic activity of compound **2** and **4b** on human OA cartilage explants at maximal concentrations of 100 and 10 µM, respectively, as at these concentrations no effect on cell viability was observed (Fig. 5D).

### Effect of compounds **2** and **4b** on Aggrecan degradation in human OA cartilage explants

**Fig. 6:**
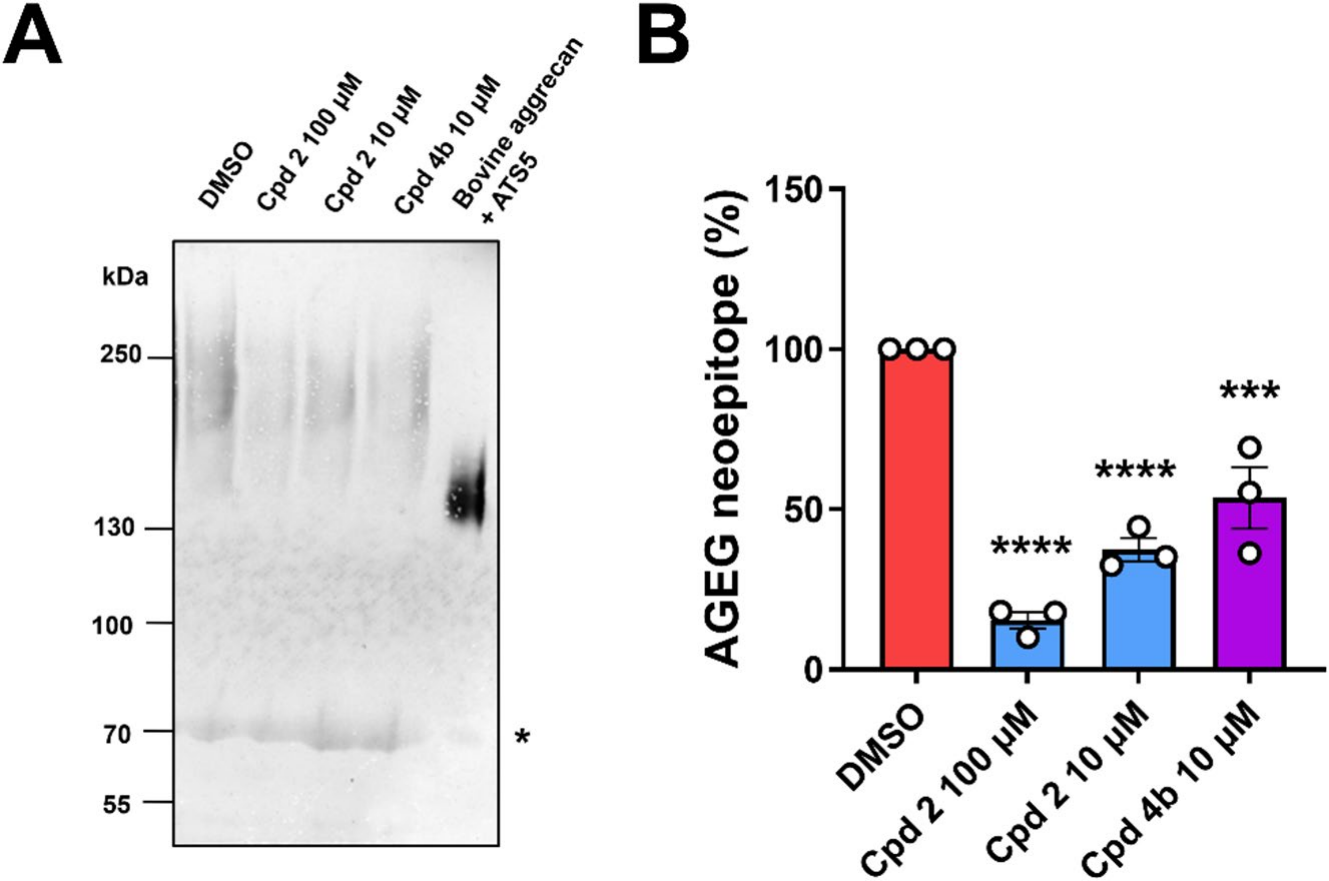
Anti-aggrecanolytic effect of compounds 2 and 4b on human OA cartilage explants. Compounds **2** and **4b** or DMSO were incubated with cartilage explants from OA patients. (**A**) Representative anti-AGEG immunoblot. ADAMTS5-digested bovine aggrecan was used as a positive control. Asterisk indicates non-specific bands. (**B**) Densitometric analysis of immunoreactive bands. Data are plotted as scatter dot plots with average ± S.E.M. (*n* = 3 donors). ***p < 0.001; ****p < 0.0001 compared to DMSO control by one-way ANOVA. ATS5, ADAMTS5; Cpd, compound.

Compounds **2** (10 and 100 µM) and **4b** (10 µM) were incubated with OA cartilage explants from patients undergoing knee surgery and the medium was analyzed by immunoblot with anti-AGEG neoepitope antibody (detecting aggrecan cleavage at E^1953^-A^1954^ in human aggrecan, UniProt ID P16112-1), as this is one of the most sensitive in detecting spontaneous aggrecan degradation in human OA cartilage explants^39^. Importantly, in this assay cartilage aggrecanase activity is largely derived from ADAMTS5^12,13,15^. In the presence of DMSO control, polydisperse bands at molecular weight above 130 kDa were detected, higher in size than those present in ADAMTS5-digested bovine aggrecan (Fig. 6A), as observed before^39^ (no purified human full-length aggrecan was commercially available to be used as a positive control). Both compounds showed significant inhibition of aggrecan degradation (Fig. 6B). Compound **2** inhibited aggrecan degradation by 63% and 85% at 10 and 100 µM, respectively, indicating dose-dependent inhibition. Compound **4b** was slightly less effective than compound **2** at 10 µM, with 46% reduction. Overall, these results suggest that both compound **2** and **4b** are effective ADAMTS5 inhibitors in human OA cartilage explants.

### Effect of compounds **2** and **4b** on OC cells

**Fig. 7:**
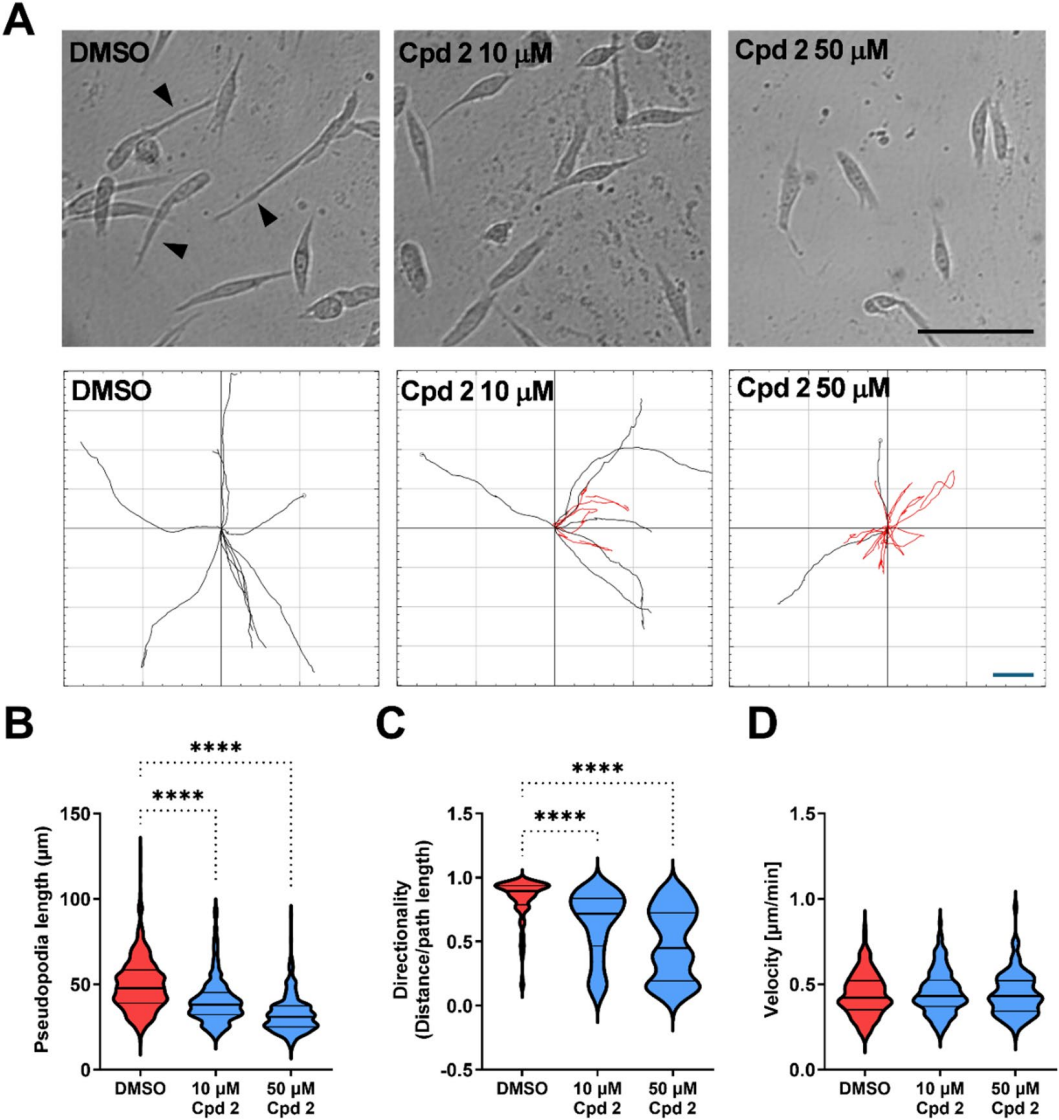
Effect of compound 2 on pseudopod elongation, directional cell migration and velocity. (**A**) A2780 OC cells over-expressing Rab25 were seeded on fibroblast-generated CDM, treated with compound **2** (10 or 50 µM) or DMSO control. Cells were imaged live with a 10x Nikon Widefield live-cell system for 16 h. Black arrows indicate elongated pseudopods. Representative single cell tracks are shown, red indicates directionality < 0.6. Bar, 100 μm. (**B**–**D**) Pseudopod length (**B**), directionality (**C**) and velocity (**D**) were measured with Fiji/ImageJ. *N* = 3 independent experiments, ****p < 0.0001 compared to DMSO control by Kruskal-Wallis test.

We have recently demonstrated that ADAMTS5 is strongly upregulated by Rab25, a small GTPase of the Ras family, and is required for OC cell migration in 2D and 3D systems^17^. We have also shown that compound **4b** effectively inhibited pseudopod elongation and directionality^17^. To test the efficacy of compound **2** in this context, we seeded Rab25 over-expressing A2780 OC cells on fibroblast-generated cell-derived matrix (CDM) (Fig. 7A). Addition of compound **2** resulted in a dose-dependent inhibition of pseudopod elongation (Fig. 7B), further supporting the role of ADAMTS5 in this process^17^. Moreover, compound **2** significantly reduced directional cell migration (Fig. 7C), without affecting the velocity of cell migration (Fig. 7D). Comparing these results with the effect we observed in the presence of **4b**^17^, compound **2** outperformed **4b** in this assay, resulting in a 35% inhibition in pseudopod elongation and a 43% inhibition in directionality of cell migration, compared to 25% and 31% upon **4b** treatment, respectively (*p* < 0.05 by Student’s t-test).

To rule out any toxicity elicited by compound **2** at the concentrations used, we assessed A2780-Rab25 cell numbers in the presence or absence of 10 µM and 50 µM compound **2**. In agreement with our data on HEK293T cells, human chondrocytes and cartilage explants (Fig. 5), we did not detect any significant difference compared to the DMSO control (**Supplementary Information Fig. ****S2**), indicating that the reduction in pseudopod elongation and direction cell migration was not due to reduced cell viability.

### Chemoinformatic analyses on compounds **2** and **4b**

The different inhibitory activity and cell toxicity profiles of compounds **2** and **4b** prompted us to undertake an extensive chemoinformatic analysis of the two molecules. In particular, to provide a rationale behind their different cytotoxic effects, we carried out: (i) analyses based on selected molecular descriptors, and (ii) extensive similarity estimations on ChEMBL compounds with reported antiproliferative activity against cell lines widely used to assess toxicities from xenobiotics (i.e., HEK293, HeLa, HepG2, SH-SY5Y and THP-1)^41–43^. These cell lines were selected also due to the lack of activity annotations on the OA-relevant chondrosarcoma cell line SW1353 (ATCC: HTB-94) in ChEMBL (**Table ****S1**). Most of the selected cell lines showed balanced numbers of annotations according to the defined activity thresholds, e.g. “Highly-active” (GI_50_, CC_50_, EC_50_, or IC_50_ < 1µM), “Active” (GI_50_, CC_50_, EC_50_, or IC_50_ between 1 µM and 10 µM), “Moderately active” (GI_50_, CC_50_, EC_50_, or IC_50_ between 10 µM and 50 µM) and “Inactive” (GI_50_, CC_50_, EC_50_, or IC_50_ > 50 µM). This distribution enabled an unbiased assessment of potential antiproliferative effects associated with compounds **4b** and **2**. The curated datasets for HEK293, HeLa, and HepG2 cells presented some overlaps (**Table ****S2**), which, however, did not influence subsequent chemoinformatic analyses. Filtered ChEMBL compounds exhibited considerable variability in the selected molecular descriptors QPlogPo/w, WPSA, Molecular Weight, NumHBD, NumHBA, Volume, QPlogBB, QPlogHERG, with descriptors for **4b** and **2** well-fitting within their recommended ranges (**Table ****S3**). This agreement in the molecular descriptors suggests that chemoinformatic approaches are appropriate for evaluating toxicity of compounds **4b** and **2** against selected cell types. We subsequently performed extensive similarity estimations using MACCS, ECFP4, and Topological Torsion (TT) fingerprints^44–46^. Using multiple types of fingerprints allowed to take into considerations different aspects of molecular similarity. In particular, MACCS fingerprints were selected to facilitate chemical interpretability among similar compounds, ECFP4-fps allowed to identify local structural variations present across scaffolds^47,48^, and TT fingerprints were employed to detect subtle variations in linear connectivity and topological features not typically captured by ECFP4 fingerprints. These analyses identified a number of compounds with ECFP4 similarity above threshold for both **4b** and **2** in the HEK293, HeLa, HepG2, SH-SY5Y and THP-1 datasets (**Table S4** and **Supplementary File 1**), suggesting that both **4b** and **2** lie within the chemical space of the curated ChEMBL datasets. However, ECFP4-based screening did not clearly discriminate among the antiproliferative profiles of **4b** and **2** toward these cell lines, and MACCS-based similarity searches did not yield compounds above threshold (**Supplementary File 1**). On the contrary, TT fingerprints provided distinct profiles for **4b** and **2** (Table 1, **Table S5** and **Supplementary File 1**). In particular, **4b** exhibited a higher degree of similarity to ChEMBL compounds classified as “Highly active” and “Active” across all the selected cell lines, while **2** showed greater similarity to compounds within the “Moderately active” or “Inactive” categories (Table 1 and **Table S5**). The chemoinformatic analyses confirmed **2** as a potentially less cytotoxic molecule, in agreement with results of calculations on molecular descriptors performed with QikProp, where most properties of **4b** and **2** fell within recommended ranges for drug-like compounds. According to the performed QikProp calculations, **2** was predicted to exhibit slightly less favorable drug-like properties than its parent compound **4b**; **4b** was predicted to show two Rule-of-Five^49^ violations, whereas **2** showed three Rule-of-Five violations. Such deviations can be frequently observed for glycosylated chemotypes and do not preclude attractive biological profiles. Indeed, glycosylation is a well-established medicinal chemistry strategy employed to improve aqueous solubility, attenuate non-specific binding, and reduce off-target liabilities. Consistent with this, the introduction of an additional monosaccharide moiety in **2** results in a marked reduction in cytotoxicity (Fig. 5), likely through a decrease in lipophilicity (**Table ****S3**), which generally lowers non-specific off-target binding. This effect is particularly relevant for kinases and enzymes possessing hydrophobic binding pockets, as well as for *h*ERG channels, which preferentially interact with more lipophilic scaffolds^50,51^. In line with this, QikProp calculations suggested that the terminal galactose moiety in **2** can improve *h*ERG-related safety profile and reduce blood-brain barrier permeability relative to its parent compound **4b** (**Table ****S3**). Furthermore, the increased polar surface area (WPSA), enhanced hydrogen-bonding capacity (NumHBD and NumHBA), and reduced lipophilicity (QPlogPo/w) of **2** (**Table ****S3**) are predicted to decrease passive membrane permeation and mitochondrial accumulation, both of which can contribute to cytotoxicity. Collectively, these results provide a rationale for the lower cytotoxicity exhibited by **2**, in agreement with our biological data (Fig. 5 and **Supplementary Information Fig. ****S2**).

**Table 1:** Number of ChEMBL compounds similar to **4b** and **2** according to similarity estimations.

Cell line	Intervals of activity	Number of compounds similar to 4b (TT-fp)	Number of compounds similar to 2 (TT-fp)
HEK293	< 1 µM	4	0
	≥ 1 to <10 µM	4	0
	≥ 10 to <50 µM	9	19
	≥ 50 µM	14	38
HeLa	< 1 µM	30	28
	≥ 1 to <10 µM	78	54
	≥ 10 to <50 µM	157	109
	≥ 50 µM	96	141
HepG2	< 1 µM	32	16
	≥ 1 to <10 µM	125	61
	≥ 10 to <50 µM	193	96
	≥ 50 µM	80	103
SH-SY5Y	< 1 µM	47	7
	≥ 1 to <10 µM	1	1
	≥ 10 to <50 µM	6	1
	≥ 50 µM	0	15
THP-1	< 1 µM	5	2
	≥ 1 to <10 µM	10	2
	≥ 10 to <50 µM	3	1
	≥ 50 µM	12	11

### Binding mode of compound **2** compared to **4b**

**Fig. 8:**
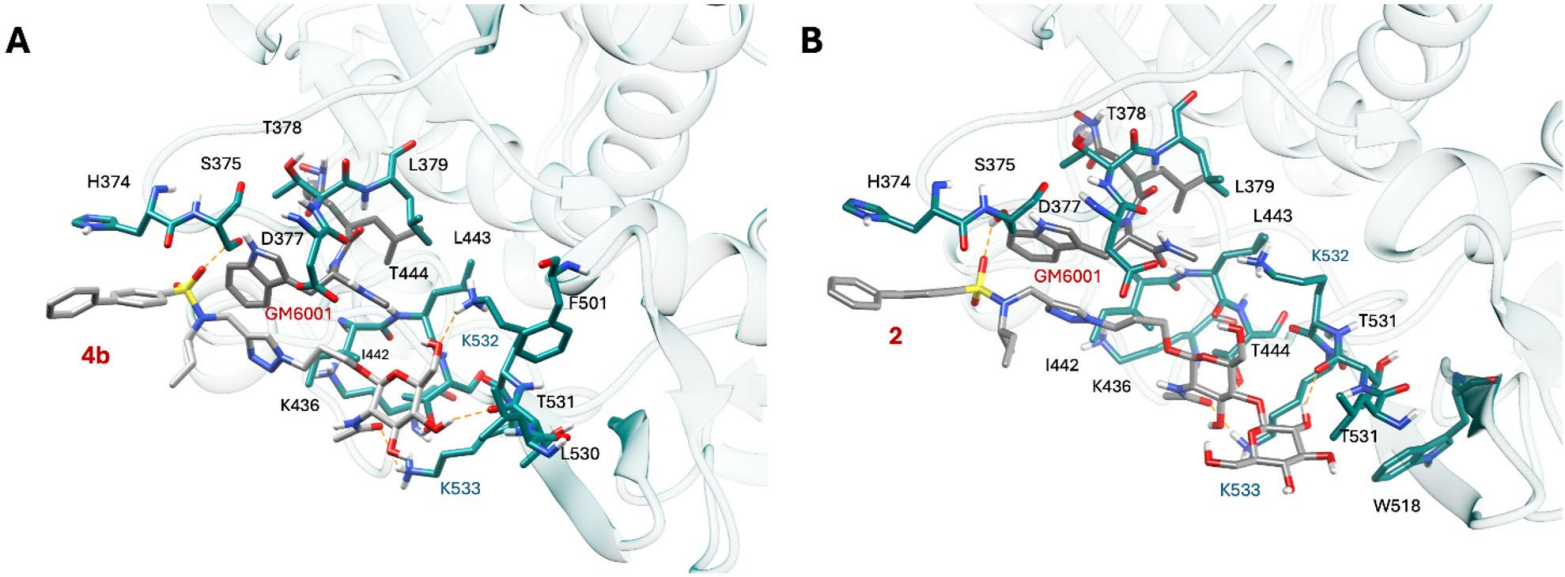
Predicted binding mode of 4b (**A**) and 2 (**B**) in ADAMTS5 metalloproteinase/Dis domain. Binding site residues are shown as deep teal sticks, while ligands are depicted as light grey sticks. H-bond interactions are depicted as light orange dashed lines, exosite residues K532 and K533 in cyan.

To compare the binding mode of compounds **2** and **4b**, both molecules were docked into ADAMTS5 (PDB ID: 2RJQ)^52^. This structure was selected for consistency with previous structure-based analyses^29^. Structure-based analyses were carried out to assess the steric and electrostatic complementarity of **4b** and **2** with the ADAMTS5 ^532^KK^533^ exosite^29^ and evaluate the impact of introducing a galactose moiety into the **4b** scaffold. Consistent with previous studies on **4b**^29^, GM6001 was first docked into the ADAMTS5 active site. The docking protocol was initially validated by successfully reproducing the crystallographic pose of the broad-spectrum inhibitor BB-94 (Batimastat)^53^ within ADAMTS5, achieving a Root-Mean-Square Deviation (RMSD) value below 2.0 Å. Subsequent docking of GM6001 into the validated docking model demonstrated a marked overlap with the co-crystallized ligand, BB-94 (**Supplementary Information Fig. ****S3**). We then proceeded to dock compounds **4b** and **2** into the ADAMTS5 ^532^KK^533^ exosite. Prior to docking, a restrained minimization of the 2RJQ-GM6001 complex was performed to refine the generated ligand-protein complex. Subsequently, the docking grid was centered near GM6001, treating it as part of the binding site complex, in agreement with earlier studies^29^. According to the predicted binding pose, one sulfonamide oxygen of **4b** establishes a H-bond interaction with the side chain of S375; the triazole ring is involved in π-π stacking interactions with the indole portion of GM6001 (Fig. 8A).

Moreover, **4b** was also predicted to make a series of H-bond interactions with the ADAMTS5 residues K532 (side chain) and T531 (backbone) by means of two of the hydroxyl groups present in its glucosamine portion; the acetyl group present in this moiety of the compound was predicted to establish a H-bond with the side chain of K533 (Fig. 8A). We have previously shown that this interaction is crucial for selectivity over ADAMTS4, as an ADAMTS5 variant where K533 was replaced by the corresponding residue in ADAMTS4 (ADAMTS5 K533H) was not inhibited by **4b**. Docking calculations on **2** predicted a H-bond interaction between one sulfonamide oxygen of the compound with the side chain of S375; moreover, a series of π-π stacking interactions were also established by the triazole ring of compound **2** with the indole portion of GM6001   (Fig. 8B), similarly to compound **4b** (Fig. 8A). However, **2** was predicted to perform only a H-bond interaction through its *N*-acetyl-glucosamine portion with the side chain of K533, while the interaction with the backbone of T531 being established by one of the hydroxyl groups of the distant galactose portion of **2** (Fig. 8B). The inability of **2** to establish a strong H-bond interaction with K533 as well as the lack of interactions with K532 may explain its lower *in vitro* inhibitory activity against ADAMTS5, compared to **4b** (Fig. 4). Although weaker than the interactions between **4b** and K533, the single H-bond between **2** and the side chain of ADAMTS5 K533 was sufficient to maintain selectivity over ADAMTS4.

Results of the docking calculations were also refined with the post-docking tool BEAR, which has demonstrated to improve docking results in a variety of virtual screening studies^48,54–56^. In line with docking results, BEAR^54^ provided binding free-energies in favor of **4b**, the predicted Molecular Mechanics and Poisson Boltzmann surface area (MM-PBSA)^54,57^ free energy scores being − 25.9 Kcal·mol^− 1^ for **4b** and − 21.6 Kcal·mol^− 1^ for **2**. Collectively, the results of these structure-based calculations confirmed **4b** as the most potent of the two investigated candidates *in vitro*.

## Conclusions

To summarize, we have reported the synthesis, *in silico* analysis, and biological activity of a new small series of arylsulfonamide glycoconjugates devoid of a ZBG as selective ADAMTS5 inhibitors. Specifically, we showed that the addition of a sugar unit to compound **4b**, while not improving ADAMTS5 inhibition *in vitro*, significantly decreased cytotoxicity on a variety of cell lines and cartilage explants. The resulting derivative **2** effectively engaged ADAMTS5 in an *ex vivo* human OA model and on OC cell organoids with activity in the high-mid micromolar range. Chemoinformatic and structure-based analysis provided a rationale behind the observed biological activity of both **2** and **4b**. Low cytotoxicity, selectivity for ADAMTS5 and efficacy on human OA cartilage explants and OC cells highlight the potential of these carbohydrate-based derivatives as promising drug candidates for pathologies where inhibition of ADAMTS5 is desirable, but further studies are required to improve their inhibitory potency for translational applications.

## Experimental section

### Chemistry

^1^H NMR spectra were recorded in appropriate solvents with a Bruker Avance III HD 400 spectrometer operating at 400 MHz. ^13^C NMR spectra were recorded with the above spectrometer operating at 100 MHz. Chemical shifts (δ) are reported in parts per million and coupling constants (*J*) are reported in hertz (Hz). ^13^C NMR spectra were fully decoupled. The assignments were made, when possible, with the aid of DEPT, COSY, HSQC experiments. The first-order proton chemical shifts, δ, were referenced to residual solvents (CDCl_3_ or CD_3_OD). The following abbreviations were used to explain multiplicities: singlet (s), doublet (d), triplet (t), double doublet (dd), broad singlet (bs), and multiplet (m). Melting points were determined on a Leica Galen III microscope (Leica/Cambridge Instruments) and were not corrected. Where indicated, the elemental compositions of the compounds agreed to within ± 0.4% of the calculated values. Chromatographic separations were performed on silica gel columns by flash column chromatography (Kieselgel 40, 0.040–0.063 mm, Merck) or using ISOLUTE Flash Si II cartridges (Biotage) or using the automated system Isolera Prime from Biotage (Uppsala, Sweden) equipped with a UV detector with a range of 200–400 wavelengths (λ). The microwave-assisted reactions were carried out in a Biotage Initiator + Microwave Synthesizer. Reactions were followed by thin-layer chromatography (TLC) on Merck aluminum silica gel (60 F_254_) sheets that were visualized under an UV lamp. Evaporation was performed *in vacuo* by rotary evaporator. Sodium sulfate was always used as the drying agent. Commercially available chemicals were purchased from Merck Life Science S.r.l. All reactions involving air- or moisture-sensitive reagents were performed under an argon or nitrogen atmosphere by using anhydrous solvents. High-resolution mass spectra (HRMS ESI) were recorded by direct injection at 5 (positive) and 7 (negative) µl min^− 1^ flow rate in an Orbitrap high-resolution mass spectrometer (Thermo, San Jose, CA, USA), equipped with HESI source. The final compounds **1–4** were synthesized with a purity of at least 95%, as confirmed by combustion analysis and one- and two-dimensional NMR analysis reported in the Supplementary Information.

#### General procedure for the synthesis of arylsulfonamide glycoconjugates **1** and **2**

In a microwave vial, 1 equiv of known azido derivative (**5** or **6**)^36,37^, 1 equiv of alkynyl derivative **7**^29^, 1 equiv of CuSO₄, and 3 equiv of sodium ascorbate were combined in a DMF/H₂O mixture (4:1). The resulting mixture was subjected to microwave irradiation for 45 min. at 100 °C (MW parameters: absorption normal, pre-stirring 1 min). The reaction mixture was treated with saturated NH₄Cl solution and extracted twice with an organic solvent (EtOAc or CHCl_3_). The organic layers were combined, dried over Na₂SO₄, filtered, and concentrated under reduced pressure. The crude product was dissolved in a solution of NH₃-MeOH 7 N and MeOH (1:1) and stirred at room temperature for 3.5 h. The mixture was then evaporated, and the resulting crude product was purified by flash chromatography to obtain the deacetylated compound (**1** or **2**) as a white solid.

*(N-((2R*,*3R*,*4R*,*5 S*,*6R)-2-((6-(4-((N-(sec-butyl)-[1*,*1’-biphenyl]-4-sulfonamido)methyl)-1H-1*,*2*,*3-triazol-1-yl)hexyl)oxy)-4*,*5-dihydroxy-6-(hydroxymethyl)tetrahydro-2 H-pyran-3-yl)acetamide) (****1****).* Starting from azido derivative **5**, after the deprotection reaction the solution was concentrated under reduced pressure, yielding crude product (217 mg), which was then purified by flash chromatography using ISOLUTE Si II 10 g cartridge (20:1 CHCl_3_/MeOH) leading to pure product **1** (176 mg, 82% yield over two steps from compound **5**). Mp: 116–118 °C. ^1^H NMR (400 MHz, CDCl_3_) *diastereoisomeric form* δ: 7.92 (s, 1H, Ar-*H triazole*), 7.93–7.89 (m, 2 H, Ar-*H*), 7.82 (m, 2 H, Ar-*H*), 7.69 (m, 2 H, Ar-*H*), 7.49 (m, 2 H, Ar-*H*), 7.41 (m, 1H, Ar-*H* triazole), 4.47 (q, *J* = 16.4 Hz, 2 H, C*H*_*2*_NSO_2_), 4.40–4.36 (m, 3 H, C*H*_*2*_N, H-1), 3.92–3.83 (m, 3 H, H-6b, C*H*N, 1×C*H*_2_O), 3.70–3.60 (m, 2 H, H-2, H-6a), 3.48–3.40 (m, 2 H, H-3, 1×C*H*_2_O), 3.33–3.23 (m, 2 H, H-5, H-4), 1.97 (s, 3 H, C*H*_*3*_CONH), 1.90–1.83 (m, 2 H, C*H*_*2*_CH_3_), 1.56–1.24 (m, 8 H, C*H*_2_C*H*_2_), 1.01 (d, *J* = 6.8 Hz, 3 H, CH_3_CH), 0.71 (t, *J* = 7.6 Hz, 3 H, CH_2_C*H*_3_). ^13^C NMR (100 MHz, CD_3_OD) *diastereoisomeric form*: δ 173.6 (*C* = O), 147.1, 146.8 (Ar-*C*-SO_2_, *C*-triazole), 141.0, 140.5 (2×Ar-*C*), 130.2, 129.6, 128.8, 128.7, 128.3, 125.7 (Ar-*C*H), 102.7 (C-1), 78.0 (C-5), 76.10 (C-3), 72.2, 70.3 (C-4, *C*H_2_O), 62.8 (C-6), 57.6 (C-2), 57.5 (*C*HNSO_2_), 51.3 (*C*H_2_NSO_2_), 38.7 (*C*H_2_N), 31.3, 30.4, 29.2, 27.0, 26.4 (4×*C*H_2_, CH_2_*C*H_3_), 23.1 (*C*H_3_CON), 18.8 (*C*H_3_CH), 11.6 (*C*H_3_CH_2_). HRMS (ESI, *m/z*) calculated for C_33_H_47_N_5_SO_8_ [M-H]^−^: 672.30726, found 672.30750. Elemental analysis calcd (%) for C_33_H_47_N_5_O_8_S: C 58.82, H 7.03, N 10.39; found: C 58.84, H 7.06, N 10.42. *(N-((2R*,*3R*,*4R*,*5 S*,*6R)-2-(3-(4-((N-(sec-butyl)-[1*,*1’-biphenyl]-4-sulfonamido)methyl)-1H-1*,*2*,*3-triazol-1-yl)propoxy)-4-hydroxy-6-(hydroxymethyl)-5-(((2 S*,*3R*,*4 S*,*5R*,*6R)-3*,*4*,*5-trihydroxy-6-(hydroxymethyl)tetrahydro-2 H-pyran-2-yl)oxy)tetrahydro-2 H-pyran-3-yl)acetamide) (****2****).* Starting from azide **6**, after deprotection reaction the crude product was purified by flash chromatography using ISOLUTE Si II 2 g cartridge (from 100% CHCl₃ to 20:1 CHCl₃/MeOH) yielding pure glycoconjugate **2** (29 mg) as a white solid, with an overall yield of 42% over two steps. Mp: 178–181 °C. ^1^H NMR (400 MHz, CD_3_OD) *diastereoisomeric form* δ: 7.97 (s, 1H, Ar-*H* triazole), 7.91 (m, 2 H, Ar-*H*), 7.82 (m, 2 H, Ar-*H*), 7.69 (m, 2 H, Ar-*H*), 7.51–7.42 (m, 3 H, Ar-*H*), 4.50–4.42 (m, 5 H, H-1’, C*H*_2_N, C*H*_2_NSO2) 4.39 (d, *J* = 8.0 Hz, 1H, H-1), 3.93–3.50 (m, 14 H, H-2, H-2’, H-3, H-3’, H-4, H-4’, H-5, H-5’, H-6a, H-6b, H-6’a, H-6’b, 1×C*H*_2_O, C*H*CH_3_), 3.48–3.34 (m, 1H, 1×C*H*_2_O), 2.18–2.09 (m, 2 H, C*H*_2_), 2.02 (s, 3 H, C*H*_*3*_CONH), 1.47–1.39 (m, 2 H, C*H*_2_CH_3_), 1.00 (d, *J* = 4.8 Hz, 3 H, C*H*_3_CH), 0.71 (bt, 3 H, CH_2_C*H*_3_). ^13^C NMR (100 MHz, CD_3_OD) *diastereoisomeric form* δ: 173.7 (C = O), 147.2 (Ar-*C*-SO_2_, *C*-triazole), 140.9, 140.4 (2×Ar-*C*), 130.2, 129.6, 129.2, 128.8, 128.7, 128.3, 126.2 (Ar-*C*H), 105.1 (C-1), 102.9 (C-1’), 80.9 (C-3), 77.1, 77.6, 74.8, 74.1, 72.6, 70.3 (C-5, C-5’, C-4, C-4’, C-3’, C-2’), 66.5 (*C*H_2_O), 62.5, 61.9 (C-6, C-6’), 57.5 (C-2), 56.6 (*C*HNSO_2_), 48.2 (*C*H_2_NSO_2_), 38.7 (*C*H_2_N), 31.5 (CH_2_), 29.1 (CH_2_CH_3_), 23.1 (*C*H_3_CONH), 18.8 (*C*H_3_CH), 11.6 (*C*H_3_CH_2_). HRMS (ESI, *m/z*) calculated for C_36_H_51_N_5_SO_13_ [M + Na]^+^: 816.30963, found 816.30890. Elemental analysis calcd (%) for C₃₆H₅₁N₅O₁₃S: C 54.47, H 6.48, N 8.82; found: C 54.51, H 6.55, N 8.86.

#### Synthesis of (*N*-((2R,3R,4R,5 S,6R)-2-(3-(4-((*N*-(sec-butyl)-[1,1’-biphenyl]-4-sulfonamido)methyl)-1 H-1,2,3-triazol-1-yl)propoxy)-6-(((tert-butyldimethylsilyl)oxy)methyl)-4,5-dihydroxytetrahydro-2 H-pyran-3-yl)acetamide) (**10**)

A solution of compound **4b** (360 mg, 0.57 mmol, 1 equiv) was dissolved in dry pyridine (2.5 ml) under a nitrogen atmosphere. TBDMSCl (344 mg, 2.28 mmol, 4 equiv) was added portionwise to the solution and the reaction was stirred at room temperature for 1 h. The mixture was diluted with DCM and sequentially washed with saturated solutions of NaHCO₃, brine, and water. The organic phase was dried over Na₂SO₄, filtered, and concentrated under reduced pressure. Residual pyridine was removed by co-evaporation with toluene. The TBDMS-protected compound **10** was obtained as a white crystalline solid (333 mg, yield 78%). ^1^H NMR (400 MHz, CDCl_3_) *diastereoisomeric form* δ: 7.92 (s, 1H, Ar-*H* triazole), 7.89 (m, 2 H, Ar-*H*), 7.73 (m, 2 H, Ar-*H*), 7.62 (m, 2 H, Ar-*H*), 7.51–7.41 (m, 3 H, Ar-*H*), 7.17 (m, 1H, N*H-*Ac), 4.47–4.38 (m, 4 H, C*H*_2_NSO_2_, C*H*_2_N), 4.27 (d, *J*_1,2_= 8.4 Hz, 1H, H-1), 3.88–3.85 (m, 3 H, C*H*CH_3_, H-6a, H6b), 3.58–3.50 (m, 1H, H-2), 3.37–3.30 (m, 3 H, H-3, H-4, H-5), 3.20–3.18 (m, 2 H, C*H*_2_O), 2.04–1.95 (m, 2 H, CH_2_), 1.93 (s, 3 H, C*H*_*3*_CONH), 1.51–1.32 (m, 2 H, C*H*_2_CH_3_), 0.98 (2×d, *J* = 6.8 Hz, 3 H, CH_2_C*H*_3_), 0.88 (s, 9 H, *t*BuSi), 0.68 (2×t, *J* = 7.2 Hz, 3 H, CHC*H*_3_), 0.06 (s, 6 H, 2×C*H*_3_Si).

#### Synthesis of (2R,3S,4R,5R,6R)-5-acetamido-6-(3-(4-((*N*-(sec-butyl)-[1,1’-biphenyl]-4-sulfonamido)methyl)-1 H-1,2,3-triazol-1-yl)propoxy)-2-(((tert-butyldimethylsilyl)oxy)methyl)tetrahydro-2 H-pyran-3,4-diyl diacetate (**11**)

Acetic anhydride (0.2 ml) was added to a solution of **10** (845 mg, 0.11 mmol, 1 equiv) in dry pyridine (0.4 ml). The mixture was stirred at 25 °C for 16 h. The reaction mixture was diluted with EtOAc and washed with water (3 × 25 ml). The organic phase was dried over Na₂SO₄, filtered, and concentrated under reduced pressure. Residual pyridine was removed by co-evaporation with toluene. The reaction yielded acetylated derivative **11** as a white powder (88 mg, 94% yield). ^1^H NMR (CDCl_3_) *diastereoisomeric form* δ: 7.92 (m, 2 H, Ar-*H*), 7.88 (m, 1H, Ar-*H*), 7.75 (m, 2 H, Ar-*H*), 7.62 (m, 2 H, Ar-*H*), 7.51–7.41 (m, 3 H, Ar-*H*), 6.50–6.21 (m, 1H, N*H*), 5.23–5.17 (m, 1H, H-3), 5.03–4.97 (m, 1H, H-4), 4.61–4.43 (m, 5 H, C*H*_2_NSO_2_, C*H*_2_N, H-1), 4.16–4.09 (m, 1H, H-2), 4.03-4.00 (m, 1H, C*H*CH_3_), 3.82–3.75 (m, 1H, 1×OC*H*_2_), 3.7–3.67 (m, 2 H, H-6a, H-6b), 3.52–3.48 (m, 1H, H-5), 2.89–2.83 (m, 1H, 1×OC*H*_2_), 2.35–2.22 (m, 2 H, C*H*_2_), 2.04 (s, 3 H, C*H*_*3*_CONH), 2.01 (s, 6 H, 2×OAc), 1.06–0.97 (m, 3 H, CH_2_C*H*_3_), 0.85 (s, 9 H, *t*-BuSi), 0.70 − 0.63 (m, 3 H, CHC*H*_3_), 0.04 (s, 6 H, 2×C*H*_3_Si).

#### Synthesis of ((2R,3 S,4R,5R,6R)-5-acetamido-6-(3-(4-((*N*-(sec-butyl)-[1,1’-biphenyl]-4-sulfonamido)methyl)-1 H-1,2,3-triazol-1-yl)propoxy)-2-(hydroxymethyl)tetrahydro-2 H-pyran-3,4-diyl diacetate) (**12**)

Compound **11** (88 mg, 0.11 mmol, 1 equiv) was dissolved in 1.8 ml of 70% aqueous acetic acid (AcOH). The mixture was stirred under reflux at 70 °C for 1.5 h. After the reaction, the solution was concentrated under reduced pressure. To remove residual solvents, a co-evaporation with toluene/DCM was done. The residue was taken up in DCM and washed with water (2 × 25 ml). The organic phase was dried over Na₂SO₄, filtered, and concentrated under reduced pressure. The reaction yielded C-6 deprotected derivative **12** (66 mg, 87% yield). ^1^H NMR (400 MHz, CDCl_3_) *diastereoisomeric form* δ: 7.91 (m, 3 H, Ar-*H*, Ar-*H*-triazole), 7.74 (m, 2 H, Ar-*H*), 7.62 (m, 2 H, Ar-*H*), 7.51–7.41 (m, 3 H, Ar-*H*), 6.41–6.34 (m, 1H, N*H*Ac), 5.28–5.22 (m, 1H, H-3), 5.02 (t, *J* = 9.2 Hz, 1H, H-4), 4.54–4.46 (m, 5 H, C*H*_2_NSO_2_, C*H*_2_N, H-1), 4.14–4.12 (m, 1H, H-2), 3.99–3.96 (m, 1H, 1×C*H*_2_O), 3.80–3.70 (m, 2 H, C*H*CH_3_, H-6a), 3.63–3.58 (m, 1H, H-6b), 3.51–3.45 (m, 1H, H-5), 3.00 (t, *J* = 8.0, 1H, 1×C*H*_2_O), 2.13–1.88 (m, 2 H, C*H*_2_), 2.05, 2.04, 2.02 (3s, each 3 H, 2×OAc, C*H*_*3*_CONH), 1.52–1.35 (m, 2 H, C*H*_*2*_CH_3_), 0.99 − 0.97 (m, 3 H, CHC*H*_*3*_), 0.69 − 0.63 (m, 3 H, CH_2_C*H*_*3*_).

#### Synthesis of (*N*-((2R,3R,4R,5 S,6R)-2-(3-(4-((*N*-(sec-butyl)-[1,1’-biphenyl]-4-sulfonamido)methyl)-1 H-1,2,3-triazol-1-yl)propoxy)-4,5-dihydroxy-6-((((2R,3R,4 S,5R,6R)-3,4,5-trihydroxy-6-(hydroxymethyl)tetrahydro-2 H-pyran-2-yl)oxy)methyl)tetrahydro-2 H-pyran-3-yl)acetamide)(**3**)

Under a nitrogen atmosphere, a solution of **12** (100 mg, 0.14 mmol, 1 equiv) and commercially available 2,3,4,6-tetra-*O*-acetyl-α-d-galactopyranosyl bromide (172 mg, 0.42 mmol, 3 equiv) was prepared in dry DCM (3.5 ml) in the presence of molecular sieves (250 mg). The mixture was stirred for 30 min at 25 °C. AgOTf, (107.6 mg, 0.4191 mmol, 3 equiv) was added at -15 °C, and the reaction was allowed to proceed for 26 h while gradually warming to 25 °C. After, pyridine (0.05 µl) was added, and the mixture was stirred for an additional 10 min at 25 °C. The reaction mixture was diluted with CHCl₃ and filtered through Celite. The organic phase was washed with 1 M Na₂S₂O₃, (1 × 50 ml), dried over Na₂SO₄, filtered, and concentrated under reduced pressure to yield 234 mg of crude product. The crude product was purified by flash chromatography on silica gel using an eluent gradient (5:1 EtOAc/hexane to 100% EtOAc), yielding a mixture of partially pure product (25 mg). The partially pure product **13** (0.14 mmol) was dissolved in methanol (1.5 ml, MeOH), and a solution of NH₃-MeOH 7 N (2.5 ml) was added. The mixture was stirred at 25 °C for 21 h. The solvent was evaporated, yielding crude product (21 mg), which was purified by two successive triturations in diethyl ether (Et₂O) at 0 °C. A pure glycoconjugate **3** (16.5 mg, white solid) was obtained with a yield of 15% over two steps starting from **12**. Mp: 87–90 °C. ^1^H NMR (400 MHz, CD_3_OD) *diastereoisomeric form* δ: 7.97 (s, 1H, Ar-*H* triazole), 7.92 (m, 2 H, Ar-*H*), 7.82 (m, 2 H, Ar-*H*), 7.69 (m, 2 H, Ar-*H*), 7.51–7.40 (m, 3 H, Ar-*H*), 4.55–4.32 (m, 5 H, H-1’, C*H*_2_N, C*H*_2_NSO_2_), 4.18 (d, *J* = 11.2, 1H, H-1), 3.93–3.65 (m, 8 H, C*H*CH_3_, H-2, H-6a, H-6b, H-6’a, H-6’b, C*H*_2_O), 3.58–3.34 (m, 7 H, H-2’, H-3, H-3’, H-4, H-4’, H-5, H-5’), 2.11–2.10 (m, 2 H, C*H*_2_), 2.03 (s, 3 H, C*H*_*3*_CONH), 1.50–1.37 (m, 2 H, C*H*_2_CH_3_), 1.00 (d, *J* = 6.4 Hz, 3 H, CHC*H*_3_). 0.70 (t, *J* = 7.02 Hz, 3 H, CH_*2*_C*H*_*3*_). ^13^C NMR (100 MHz, CD_3_OD) *diastereoisomeric form* δ: 173.9 (C = O), 146.8, (Ar-*C*-SO_2_, *C*-triazole), 140.9, 140.4 (2×Ar-*C*), 130.2, 129.6, 129.2, 128.8, 128.7, 128.3, 127.8, 126.1 (Ar-*C*H), 105.5 (C-1), 102.8 (C-1’), 77.0, 76.7, 75.8, 75.0, 72.6, 72.1, 70.3, 70.0 (C-5, C-5’, C-4, C-4’, C-3, C-3’, C-2’, C-6’), 66.6 (*C*H_2_O), 62.5 (C-6), 57.5, 57.3 (C-2), 54.8 (*C*HNSO_2_), 48.1 (*C*H_2_NSO_2_), 38.7 (*C*H_2_N), 31.5 (CH_2_), 29.1 (*C*H_2_CH_3_), 23.2 (*C*H_3_CON), 18.9, 18.8 (*C*H_3_CH), 11.6 (*C*H_3_CH_2_). HRMS (ESI, *m/z*) calculated for C_36_H_51_N_5_SO_13_ [M + H]^+^: 794. 32768, found 794.32727, [M + Na]^+^: 816.30963, found 816.30914. Elemental analysis calcd (%) for C₃₆H₅₁N₅O₁₃S: C 54.47, H 6.48, N 8.82; found: C 54.49, H 6.52, N 8.89.

#### *Synthesis of* (*N-((2R*,*3R*,*4R*,*5 S*,*6R)-2-(3-(4-((N-(sec-butyl)-[1*,*1’-biphenyl]-4-sulfonamido)methyl)-1 H-1*,*2*,*3-triazol-1-yl)propoxy)-6-((((2R*,*3R*,*4R*,*5 S*,*6R)-3*,*4-dihydroxy-6-(hydroxymethyl)-5-(((2 S*,*3R*,*4 S*,*5R*,*6R)-3*,*4*,*5-trihydroxy-6-(hydroxymethyl)tetrahydro-2 H-pyran-2-yl)oxy)tetrahydro-2 H-pyran-2-yl)oxy)methyl)-4*,*5-dihydroxytetrahydro-2 H-pyran-3-yl)acetamide)(****4****)*

Under a nitrogen atmosphere, a solution of **12** (66 mg, 0.09 mmol, 1 equiv) and peracetyl lactose-1-α-trichloroacetoimidate (144 mg, 0.18 mmol, 2 equiv) was prepared in dry DCM (1.0 ml) in the presence of molecular sieves (50 mg). The mixture was stirred for 30 min at 25°C. A solution of 10% BF_3_-Et_2_O in DCM, (22 µL, 0.018 mmol, 0.2 equiv) was added at -15°C, and the reaction was allowed to proceed for 12 h while gradually warming to 25°C. The reaction mixture was evaporated, and the resulting crude product was purified by flash chromatography using ISOLUTE Si II 10 g cartridge (30:1 CHCl_3_/MeOH), yielding of partially pure derivative **14** (107 mg), used in the next step without any further purification. Compound **14** was dissolved in MeOH (1.5 ml), a solution of NH₃-MeOH 7 N (1.5 ml) was added and the mixture was stirred at 25°C for 5 h. The solvent was evaporated, yielding crude product (85 mg), which was purified by reverse phase flash chromatography (in gradient from 9:1 MeOH/H_2_O to 100% MeOH) by Isolera Biotage automated chromatographer using Sfar Ultra C18 6 g cartridge affording pure derivative **4** (35 mg, white solid) with a yield of 40% over two steps starting from **12**. Mp: 97–100°C. ^1^H NMR (400 MHz, CD_3_OD) *diastereoisomeric form* δ: 7.96 (s, 1H, Ar-*H* triazole), 7.91 (m, 2H, Ar-*H*), 7.83 (m, 2H, Ar-*H*), 7.70 (m, 2H, Ar-*H*), 7.53–7.46 (m, 2H, Ar-*H*), 7.45–7.39 (m, 1H, Ar-*H*), 4.57–4.32 (m, 6H, H-1’, H-1’’, C*H*_2_N, C*H*_2_NSO_2_), 4.17 (d, *J* = 11.5, 1H, H-1), 3.94–3.62 and 3.62–3.33 (2m, 21H, C*H*CH_3_, H-2, H-2’, H-2’’, H-3, H-3’, H-3’’, H-4, H-4’, H-4’’, H-5, H-5’, H-5’’, H-6a, H-6b, H-6’a, H-6’b, H-6’’a, H-6’’b, C*H*_2_O), 2.21–2.05 (m, 2H, C*H*_2_), 2.02 (s, 3H, C*H*_*3*_CONH), 1.55–1.36 (m, 2H, C*H*_2_CH_3_), 1.01 (d, *J* = 6.4 Hz, 3H, CHC*H*_3_), 0.71 (t, *J* = 7.02 Hz, 3H, CH_*2*_C*H*_*3*_). ^13^C NMR (100 MHz, CD_3_OD) *diastereoisomeric form* δ: 174.0, 163.3 (C = O), 147.2 (Ar-*C*-SO_2_, *C*-triazole), 135.6, 134.4, 131.5 (Ar-*C*), 132.7, 132.5, 130.4, 126.0, 118.0, 117.7, 116.3, 115.5, 115.2 (Ar-*C*H), 102.8 (C-1, C-1’, C-1”), 78.0, 75.9, 72.1, (C-5, C-5’, C-5’’, C-4, C-4’, C-4’, C-3, C-3’, C-3’’, C-2’, C-2’’), 68.3 (C-6), 66.6 (*C*H_2_O), 62.5 (C-6’, C-6’’), 57.4 (C-2), 49.9 (*C*HNSO_2_), 48.1 (*C*H_2_NSO_2_), 38.6 (*C*H_2_N), 31.5 (CH_2_), 29.1 (*C*H_2_CH_3_), 23.1 (*C*H_3_CON), 18.8, 18.6 (*C*H_3_CH), 11.5 (*C*H_3_CH_2_). HRMS (ESI, *m/z*) calculated for C_42_H_61_N_5_O_18_S [M + Na]^+^: 978.36245, found: 978.36157. Elemental analysis calcd (%) for C₄₂H₆₁N₅O₁₈S: C 52.77, H 6.43, N 7.33; found: C 52.80, H 6.46, N 7.30.

### Solubility determination of compounds **4b** and **2**

The solubility of compounds **4b** and **2** was assessed in PBS at pH 7.4 and room temperature. Stock solutions of the compounds (10 mM in DMSO) were diluted in PBS buffer to achieve final concentrations between 1,000 µM − 12.5 µM. Two-hundred microliters of the proper compound solutions in PBS buffer were added in a 96-well plate, and the absorbance was measured at 284 nm using a SPECTROstar Nano UV-Vis spectrophotometer (220–1,000 nm, Ortenberg, Germany). Analyses were performed in triplicate, and the mean value was calculated for each sample.

### *In silico* analyses

#### Chemoinformatic analysis

To identify potential toxicity issues associated with compounds **4b** and **2**, extensive *in silico* analyses were performed using QikProp (Schrödinger 2024-2), and 2D similarity estimations against selected datasets of ChEMBL compounds. Compounds **4b** and **2** were initially prepared with LigPrep (Schrödinger 2024-2)^58^ to generate their predominant tautomeric and ionization states under physiological conditions and minimize their structure. Subsequently, 2D similarity estimations between compounds **4b** and **2** and molecules with reported antiproliferative activity against HEK293, HeLa, HepG2, SH-SY5Y and THP-1 cells were performed. These cell lines are frequently used as cellular models for safety screenings^41–43^. Compounds with reported antiproliferative activity against the selected cell lines were firstly retrieved from ChEMBL (https://www.ebi.ac.uk/chembl)^59^ and filtered to retain only records with activity annotations with “*Standard Type*” expressed as GI_50_, CC_50_, EC_50_, and IC_50_, and “*Standard Relation*” equal to “<”, “>”, or “=”. Activity data were subsequently standardized into nanomolar units, and duplicate entries resulting from multiple assays under different experimental conditions were removed (**Supplementary File 2**). Datasets curation was performed with a custom script leveraging the Pandas library (https://pandas.pydata.org/). Filtered compounds were then prepared for the following analyses with LigPrep (Schrödinger 2024-2), using preparation settings analogous to those used for **4b** and **2**. Key molecular properties related to compounds drug-likeness were computed using QikProp^60^ and compared with the corresponding property ranges of approved drugs. Ligand similarity was performed with default settings using three different types of fingerprints (i.e., MACCS, ECFP4 and Topological Torsion, TT) implemented in the RDKit python library (https://www.rdkit.org/). The degree of similarity between **4b** and **2**, and previously curated ChEMBL compounds was evaluated in terms of Tanimoto score. Similarity records were filtered to retain only pairs of molecules showing similarity scores higher than 0.8 (Tanimoto_MACCS_), 0.3 (Tanimoto_ECFP4_) and 0.279 (Tanimoto_TT_), which are commonly used thresholds in ligand-based screenings^61,62^. A tailored KNIME workflow^63^ was devised and applied in order to evaluate the statistics related to the calculated similarities.

#### Structure-based analysis

Docking calculations were performed with the Glide docking program (Schrödinger). The ADAMTS5 PDB structure 2RJQ^52^ was prepared for docking by using the *Protein Preparation Wizard* module available in the Maestro (Schrödinger 2024-2) suite^64^. Missing side chains and hydrogens were firstly added, and the ionization and tautomerization states of the protein residues adjusted according to physiological conditions. The structure was refined with a restrained minimization employing a RMSD of 0.30 Å relative to the input protein coordinates. To ensure consistency with previously reported docking studies of **4b**^29^, GM6001 was first docked into the ADAMTS5 binding site (**Supplementary Information Fig. ****S3**). Docking calculations were performed using default parameters of the standard precision (SP) protocol available in Glide (Schrödinger)^65^, with the docking grid centered on the co-crystallized ligand BB-94/Batimastat (PDB HET ID: BAT) of the 2RJQ structure^52^. The docking accuracy was confirmed by redocking BB-94 into its active site. Given prior evidence that **4b** occupies a region proximal to GM6001^29^, the docking calculations of **4b** and **2** were performed with a grid centered near the docked GM6001, specifically encompassing residues H374, S375, H410, H414, K532, and K533. These docking calculations were performed with the SP protocol of Glide (Schrödinger), using the “enhanced sampling” setting for conformer generation and the “expanded sampling” for the selection of the initial poses. A visual inspection of the generated poses was performed for evaluating protein-ligand complexes of **4b** and **2**, as previously observed^29^. The generated poses were then further refined with the BEAR post-docking processing tool (default settings)^54^.

### Biological studies

#### Protein expression and purification

Human recombinant full-length FLAG-tagged ADAMTS4 and ADAMTS5 were expressed in HEK293T cells (Thermo Fisher Scientific) and purified by anti-FLAG immunoaffinity chromatography as before^29^. Concentrations of active proteases were determined by active-site titrations with known concentrations of TIMP-3 (Bio-Techne, 973-TM-010), using QF peptides as substrates^66^. V1-5GAG, a truncated version of versican V1 isoform comprising residues 21–694 and containing a C-terminal 6x His tag^67^, was expressed in HE293T cells and purified by immobilized metal affinity chromatography as before^30^.

#### QF peptide cleavage assays

QF peptide cleavage assays were conducted in TNC-B (50 mM Tris–HCl, pH 7.5, 150 mM NaCl, 10 mM CaCl_2_, and 0.02% NaN_3_) on a SpectraMax i3x Multi-Mode Platform (Molecular Devices, UK) in a total volume of 20 µl at 37℃ as before^29^. ADAMTS4 (3.5 nM) or ADAMTS5 (4 nM) were incubated with compounds **1–4** (100 µM, diluted in TNC-B from 10 mM DMSO stocks) or DMSO for 1 h at 37 ℃ before addition of QF peptides fluorescein-5(6)-carbonyl-Ala-Glu-Leu-Asn-Gly-Arg-Pro-Ile-Ser-Ile-Ala-Lys-N, N,N0,N0-tetramethyl-6-carboxyrhodamine, and fluorescein-5(6)-carbonyl-Thr-Glu-Ser-Glu - Ser-Arg-Gly-Ala-Ile-Tyr-Lys-Lys-N, N,N0,N0-tetramethyl-6-carboxyrhodamine, respectively, each at 40 µM. Fluorescence intensity was recorded with an excitation wavelength of 485 nm and an emission wavelength of 535 nm every min for 2 h, expressed as relative fluorescence units and normalized against a blank containing only buffer and substrate. Fluorescence values were converted into residual activity by fixing as 100% the activity of the reactions containing an equal concentration (v/v) of DMSO instead of compound (DMSO control).

#### Versicanase assay

Compounds **1–4** (100 µM) or DMSO were incubated with ADAMTS4 (5 nM) or ADAMTS5 (0.4 nM) for 2 h at 37 °C, before addition of V1-5GAG (50 nM). At each time point, reactions were stopped by addition of ethylenediaminetetraacetic acid and ADAMTS-generated versican fragments (versikine) quantified by sandwich ELISA as before^29,30,38^ using anti-DPEAAE neoepitope antibody (Life Technologies, PA1-1748 A, 5 µg/ml) and polyclonal anti-versican antibody (Abcam, ab171887, 3 µg/ml). For each reaction, the amount of versikine generated was derived from a standard curve (0–1.56 nM) of V1-5GAG completely digested with ADAMTS5. Initial velocities were calculated from the concentration of versikine generated as a function of reaction time. Percent of inhibition was calculated from the DMSO control reactions. IC_50_ values were determined using the formula: *v*_i_/*v*_0_ = 1/(1 + [I]/IC_50_) where *v*_i_ is the initial velocity of substrate cleavage in the presence of the inhibitor at concentration [I] and *v*_0_ is the initial velocity in the presence of an equal concentration (v/v) of DMSO.

#### Aggrecanase assay

Aggrecanase assays were carried out as before^29^. ADAMTS5 (1 nM) was incubated with compounds **2** and **3** or DMSO for 2 h at 37 °C in TNC-B buffer before addition of bovine articular aggrecan (UniProt ID: P13608, 270 nM) (Merck, A1960). After 2 h digestion at 37 °C, the reactions were stopped with ethylenediaminetetraacetic acid and the samples incubated with 0.1 U/ml of chondroitinase ABC (AMS Biotechnology, Abingdon, UK) and keratanase (endo-beta galactosidase, Merck, G6920) in deglycosylation buffer (50 mM sodium acetate, 25 mM Tris HCl pH 8.0) for 16 h at 37 °C to remove GAG chains.

#### SDS PAGE and immunoblots

Samples were separated by SDS-PAGE under reducing conditions (5% β-mercaptoethanol) on BOLT, 4–12%, Bis-Tris Gels (Invitrogen) in MES Running Buffer (NuPAGE). Proteins were transferred onto a nitrocellulose membrane using Trans-Blot Turbo™ Transfer System (BioRad), at 25 V, 2.5 A for 30 min. Membranes were blocked with 3% BSA/PBS for 1 h at room temperature. Aggrecan fragments generated by ADAMTS cleavage at E^392^-A^393^^8^ or E^1953^-A^1954^^68^ were detected using mouse monoclonal anti-ARGSV antibody (BC3, Life Technologies, MA3-16888, 4 µg/ml) or polyclonal anti-AGEG neoepitope antibody (serum, 1:200 dilution)^12,33^, respectively, followed by the appropriate horseradish peroxidase (HRP)-conjugated secondary antibodies (polyclonal Goat Anti-Mouse P0447, 0.1 ng/µl and polyclonal Goat Anti-Rabbit, P0448, 0.03 ng/µl, both from Agilent Technologies LDA UK Ltd). All antibodies were diluted in 0.5% bovine serum albumin (BSA)/PBS. Washes between each step were carried out in triplicate, for 15 min each, with PBS-Tween (0.1%). Following addition of the chemiluminescent HRP substrate (Millipore, WBKL30500) membranes were imaged using Bio-Rad Chemidoc Touch Imaging System. Analysis was carried out using BioRad ImageLab software (version 5.2.1) using sequential exposures to avoid saturation artifacts^69^. Figures for unprocessed blots are included in **Supplementary information Fig. S4** and **Fig. S5** with highlighted regions (in red boxes) corresponding to the data reported in the main manuscript figures.

#### Cartilage explant cultures and chondrocytes

Porcine articular cartilage from the joints of 3–9 month old pigs was supplied by a local abattoir within 2 h of slaughter and dissected into small pieces approximately 3 mm long and 3–4 mm wide. Each piece weighed roughly 10 mg. After dissection, the cartilage was allowed to acclimatize for 24 h at 37 °C under 5% CO_2_ in Dulbecco’s Modified Eagle Medium (DMEM) containing 100 U/ml penicillin, 100 µg/ml streptomycin, 10% fetal bovine serum (FBS).

Human OA cartilage was obtained from Clatterbridge Hospital, Wirral, UK, following total knee arthroplasty procedures with informed patient consent in full compliance with national and institutional ethical requirements, the United Kingdom Human Tissue Act, and the Declaration of Helsinki (REC 18/WA/0344). Explants (~ 36 mm^3^, ~ 40 mg wet volume/weight) from each of 3 donors were placed in one well of a 48-well plate and allowed to rest for 24 h in 1 ml of DMEM containing 10% FBS before use. The medium was replaced, and the cartilage was rested for a further 24 h in 1 ml DMEM at 37 °C before assays.

Non-OA human chondrocytes were isolated from knee cartilage tissues obtained from the Stanmore BioBank, Institute of Orthopaedics, Royal National Orthopaedic Hospital, Stanmore, UK, from 3 donors following informed consent and approval by the Royal Veterinary College Ethics and Welfare Committee (Institutional approval URN 2012 0048 H) as before^70^.

#### Detection of Aggrecan fragments released from human OA cartilage

Explants were incubated in 0.2 ml of fresh DMEM containing compound **2** (10 or 100 µM) or **4b** (10 µM) for 48 h. The medium was collected and deglycosylated as above. Proteins were precipitated using ice-cold acetone, dissolved in the SDS-sampling buffer and then analyzed by immunoblot using anti-AGEG aggrecan neoepitope antibody as before^12^.

#### Cell viability assays

Cell viability was assessed using CellTiter^®^ 96 Aqueous One Solution Cell Proliferation Assay (Promega, G3582) following the manufacturer’s protocol. The assay is based on the reduction of [3-(4,5-dimethylthiazol-2-yl)-5-(3-carboxymethoxyphenyl)-2-(4-sulfophenyl)-2 H-tetrazolium] (MTS) and phenazine ethosulfate into formazan. Compounds **2** and **4b**, GM6001 (Merck, 364206) (10–100 µM), or DMSO control were incubated in 48-well Nunc™ plates (Thermo Fisher Scientific,150687) with HEK293T cells, human non-OA chondrocytes, porcine cartilage explants, or human OA cartilage explants for 72 h. At least 3 independent experiments were carried out with 3 technical replicates each. For experiments involving human samples, independent experiments were carried out on 3 different donors. Absorbance at 490 nm was measured on a SpectraMax i3x Multi-Mode Platform (Molecular Devices). Cell viability was normalized to DMSO controls.

#### Assays on OC cells

##### Cell culture

A2780 OC cells over-expressing Rab25 (A2780-Rab25), generated in G. Mills lab^71^, and telomerase-immortalised human fibroblasts (TIFs) were a gift from Jim Norman lab (CRUK Scotland Institute). A2780-Rab25 cells were grown in RPMI media (Gibco), supplemented with 10% (v/v) FBS. TIFs were grown in DMEM, supplemented with 10% FBS. To maintain Rab25 over-expression, A2780-Rab25 cells were selected with 0.4 mg/ml G418 every 10 passages.

##### CDM generation

CDMs were generated as previously described^72^ in 12 well plates. Wells were coated with 0.2% gelatin for 60 min at 37º C, crosslinked with 1% glutaraldehyde for 30 min at room temperature, quenched with 1 M glycine for 20 min at room temperature and equilibrated in DMEM for 30 min at 37 °C. TIFs (2.5 × 10^5^ per well) were seeded for 24 h to achieve confluency and grown in the presence of 50 µg/ml ascorbic acid for 9 days. CDMs were washed with PBS, incubated with extraction buffer (20 mM NH_4_OH, 0.5% Triton X-100 in PBS with Ca^2+^ and Mg^2+^) for ~ 2 min, until no intact cells were visible by phase, washed twice with PBS with Ca^2+^ and Mg^2+^ and treated with 15 µg/ml DNase for 1 h at 37º C, 5% CO_2_. CDMs were washed twice with PBS with Ca^2+^ and Mg^2+^ and stored in PBS with Ca^2+^ and Mg^2+^ at 4º C for up to one month.

##### OC cell migration

A2780-Rab25 cells (5 × 10^4^ per well) were seeded onto a CDM-coated 12-well plate. Compound **2** was added 4 h after plating. Plates were imaged in a Nikon Inverted Ti eclipse microscope with Oko-lab environmental control chamber with a 10x/NA 0.45 objective. Cells were incubated at 37 °C and 5% CO_2,_ images were acquired every 10 min for 12 h and more than 40 cells per well were quantified per biological replicate. Individual cell migration was manually tracked using MTrack2, a Fiji/ImageJ plugin. The chemotaxis tool plugin in Fiji/Image J (https://ibidi.com/chemotaxis-analysis/171-chemotaxis-and-migration-tool.html) was used to calculate the velocity and directionality of migrating cells.

##### OC cell proliferation

A2780-Rab25 (3,000 cells per well) were seeded in a 96-well plate and incubated with compound **2** (10 µM and 50 µM) or DMSO control for 24 h. Cells were fixed with 4% paraformaldehyde and stained with 5 µM DRAQ5 in PBS for 1 h at room temperature with gentle rocking. Cells were washed twice with PBS for 30 min and kept in PBS for imaging. DRAQ7 was detected by the 700 nm channel (200 μm resolution) of a Licor Odyssey Sa instrument. The signal intensity (total intensity minus total background) of each well was quantified in Image Studio Lite software.

#### Statistical analysis

Statistical analysis was performed on GraphPad Prism software (version 8.4). Data was tested for normality using Shapiro-Wilk test. For comparison between 2 groups, Student’s t-test was used. For comparisons between more than 2 groups, one-way ANOVA or Kruskal-Wallis test were used for normally and non-normally distributed data, respectively. *p* < 0.05 was considered statistically significant.

## Supplementary Information

Below is the link to the electronic supplementary material.

Supplementary Material 1

Supplementary Material 2

Supplementary Material 3

## Data Availability

The authors declare that the data supporting the findings of this study are available within the paper and its Supplementary Information files. Should any raw data files be needed in another format they are available from the corresponding authors upon reasonable request.

## Abbreviations

ADAMTS: A Disintegrin-like And Metalloprotease domain with Thrombospondin type I motifs
CDM: Cell-derived matrix
Dis: Disintegrin-like domain
DMF: Dimethylformamide
GAG: Glycosaminoglycan
NMR: Nuclear magnetic resonance spectroscopy
OC: Ovarian cancer
OA: Osteoarthritis
QF: Quenched-fluorescent
TIF: Telomerase-immortalised human fibroblast
ZBG: Zinc-binding group
