# Supplementary material for: Design, synthesis and biological activity of glycoconjugated ADAMTS5 exosite inhibitors: applications in osteoarthritis and ovarian cancer models
Source: Sci Rep. 2025 Dec 11;16:329. doi: 10.1038/s41598-025-29549-3 (PMC12769565; doi:10.1038/s41598-025-29549-3)
Supplement: Supplementary file 3 — Supplementary Material 3 [file 41598_2025_29549_MOESM3_ESM.docx]

**SUPPLEMENTARY INFORMATION**

**Design, synthesis and biological activity of glycoconjugated ADAMTS5 exosite inhibitors: applications in osteoarthritis and ovarian cancer models**

Doretta Cuffaro^1^, Sophie Blagg^2^, Kazuhiro Yamamoto,^3,4^ Luca Pinzi,^5^ Rachele Bacchetti, ^6^ Shengnan Yuan,^6^ Simon Tew,^3^ Paola Campagnolo,^7,8^ Felicia D’Andrea,^1^ Enrico Crispino,^1^ Giulio Rastelli,^5^ Armando Rossello,^1^ Elena Rainero,^6^ Elisa Nuti^1^,* and Salvatore Santamaria^2,7*^

^1^Department of Pharmacy, University of Pisa, via Bonanno 6, 56126 Pisa, Italy

^2^Department of Immunology and Inflammation, Imperial College London, Du Cane Road, W12 0NN, London, United Kingdom;

^3^Institute of Life Course and Medical Sciences, University of Liverpool, L7 8TX, Liverpool, United Kingdom;

^4^Faculty of Agriculture, Scleroprotein and Leather Research Institute, Faculty of Agriculture, Tokyo University of Agriculture and Technology, 3-5-8  Saiwaicho, Fuchu, Tokyo 183-8509, Japan;

^5^Department of Life Sciences, University of Modena and Reggio Emilia, Via Giuseppe Campi, 103. 41125 Modena, Italy;

^6^School of Biosciences, University of Sheffield, Western Bank, S10 2TN, United Kingdom;

^7^Discipline of Clinical Sciences, School of Biosciences, University of Surrey, Guildford, Surrey GU2 7XH, United Kingdom;

^8^Department of Comparative Biomedical Sciences, School of Veterinary Medicine, Guildford, Surrey GU2 7XH, United Kingdom;

* to whom the correspondence should be addressed: Elisa Nuti, Department of Pharmacy, University of Pisa, via Bonanno 6, 56126 Pisa, Italy; [elisa.nuti@unipi.it](mailto:elisa.nuti@unipi.it); Salvatore Santamaria, ^2^Department of Immunology and Inflammation, Imperial College London, Du Cane Road, W12 0NN, London, United Kingdom; [s.santamaria@imperial.ac.uk](mailto:s.santamaria@imperial.ac.uk); Discipline of Clinical Sciences, School of Biosciences, University of Surrey, Guildford, Surrey GU2 7XH United Kingdom; [s.santamaria@surrey.ac.uk](mailto:s.santamaria@surrey.ac.uk);

**Table of Contents**

**Tables**

Table S1….………………………………………………………………………………….. S3

Table S2….………………………………………………………………………………….. S4

Table S3….………………………………………………………………………………….. S5

Table S4….………………………………………………………………………………….. S6

Table S5….………………………………………………………………………………….. S7

**Figures**

Figure S1....………………………………………………………………………………….. S8

Figure S2....………………………………………………………………………………….. S8

Figure S3…………………………………………………………………………………….. S9

Figure S4………………………………………………………………………………….... S10

Figure S5………………………………………………………………………………..….. S10

NMR spectra of final compounds (**1**, **2**, **3** and **4**) ....………………………………………... S11

HRMS spectra of final compounds (**1**, **2**, **3** and **4**)………………………………………….. S20

**Table S1**: Number of molecules with activity records on HEK293 (CHEMBL614818), HepG2 (CHEMBL395), HeLa (CHEMBL399), SH-SY5Y (CHEMBL614910), SW1353 (CHEMBL614944) and THP-1 (CHEMBL614245) cell lines retrieved from ChEMBL, after data curation.

| **Target Name** | **Number of compounds (Total)** | **Number of compounds**  **(< 10 µM)** | **Number of compounds (10µM-50 µM)** | **Number of compounds**  **(> 50 µM)** |
| --- | --- | --- | --- | --- |
| HEK293 | 4,039 | 882 | 1,472 | 1,685 |
| HepG2 | 24,625 | 9,068 | 9,565 | 5,992 |
| SH-SY5Y | 1,425 | 486 | 511 | 428 |
| SW1353 | 2 | 2 | 0 | 0 |
| HeLa | 27,270 | 11,852 | 9,042 | 6,376 |
| THP-1 | 2,390 | 843 | 680 | 867 |

**Table S2**: Number of compounds showing activity annotations on the selected cell lines.

| **Cell line** | **Number of compounds with activity annotations per cell line(s)** |
| --- | --- |
| HEK293 | 2,197 |
| HepG2 | 18,067 |
| HeLa | 21,147 |
| THP-1 | 1,905 |
| SH-SY5Y | 830 |
| HeLa, HepG2 | 4,920 |
| HEK293, HepG2 | 852 |
| HEK293, HeLa | 557 |
| HepG2, SH-SY5Y | 190 |
| HepG2, THP-1 | 178 |
| HeLa, THP-1 | 165 |
| HEK293, SH-SY5Y | 164 |
| HeLa, SH-SY5Y | 83 |
| HEK293, THP-1 | 19 |
| SH-SY5Y, THP-1 | 1 |
| HEK293, THP-1 | 19 |
| HEK293, HeLa, HepG2 | 171 |
| HeLa, HepG2, SH-SY5Y | 105 |
| HeLa, HepG2, THP-1 | 73 |
| HEK293, HepG2, SH-SY5Y | 24 |
| HEK293, HeLa, THP-1 | 9 |
| HEK293, HepG2, THP-1 | 9 |
| HEK293, HeLa, SH-SY5Y | 6 |
| HepG2, SH-SY5Y, THP-1 | 2 |
| HEK293, HeLa, HepG2, THP-1 | 14 |
| HEK293, HeLa, HepG2, THP-1 | 14 |
| HEK293, HeLa, HepG2, SH-SY5Y | 5 |
| HeLa, HepG2, SH-SY5Y, THP-1 | 3 |
| HEK293, HeLa, HepG2, SH-SY5Y, THP-1 | 12 |

**Table S3**: **Molecular descriptors of compounds 4b and 2 as calculated with QikProp**. The (max-min) range of values of the same molecular descriptor evaluated for ChEMBL compounds with activity annotations on the selected cell lines ligands; **4b** and **2** are also reported for comparison. Values outside recommended ranges are highlighted in bold for **4b** and **2**. Recommended value ranges according to the QikProp (Schrödinger 2024-2) manual were the following: QPlogPo/w (predicted octanol/water partition coefficient): -2.0 – 6.5; WPSA (weakly polar component of the SASA (i.e., halogens, P, and S)): 0.0 – 175.0; MW (molecular weight): 130.0 – 725.; NumHBD (estimated number of hydrogen bonds that would be donated by the compound): 0 – 6; NumHBA (Estimated number of hydrogen bonds that would be accepted by the compound): 0 – 20; Volume (total solvent-accessible volume in cubic angstroms): 500 – 2000; QlogBB (predicted brain/blood partition coefficient): -3.0 – 1.2; QPlogHERG (predicted IC_50_ value for blockage of HERG K^+^ channels): concern below -5.0.

| **Target Name** | **QPlogPo/w (Max - Min)** | **WPSA**  **(Max - Min)** | **MW**  **(Max - Min)** | **NumHBD**  **(Max - Min)** | **NumHBA (Max - Min)** | **Volume**  **(Max - Min)** | **QPlogBB**  **(Max - Min)** | **QPlogHERG**  **(Max - Min)** |
| --- | --- | --- | --- | --- | --- | --- | --- | --- |
| 4b | 1.3 | **176.3** | 631.3 | 4 | 11 | 1,919.6 | **-3.5** | **-6.0** |
| 2 | -0.9 | **255.5** | **793.3** | **7** | 16 | **2,112.4** | **-4.4** | -4.3 |
| HEK293 | 14.5 - -21.0 | 1481.4 - 0.0 | 3947.0 - 78.0 | 51 - 0 | 54 - 0 | 10,643.46 - 318.438 | 1.2 - -66.3 | 34.6 - -13.5 |
| HepG2 | 19.9 - -20.1 | 2749.7 - 0.0 | 6658.1 - 85.0 | 101 - 0 | 84 - 0 | 14,029.919 - 317.071 | 1.5 - -131.8 | 41.8 - -14.3 |
| SH-SY5Y | 8.9 - -17.0 | 1186.9 - 3.2 | 3138.4 - 130.0 | 45 - 0 | 47 - 1 | 7,666.746 - 411.26 | 1.1 - -51.9 | 36.7 - -10.9 |
| SW1353 | 4.6 - 3.6 | 174.0 - 80.1 | 518.1 - 435.2 | 6 - 1 | 10 - 6 | 1,415.229 – 1,338.81 | -1.0 - -3.4 | -5.6 - -6.1 |
| HeLa | 20.1 - -18.9 | 1614.1 - 0.0 | 3947.0 - 88.1 | 64 - 0 | 54 - 0 | 2,334.219 - 411.26 | 0.0 - -2.9 | -2.1 - -8.0 |
| THP-1 | 16.2 - -8.2 | 1514.3 - 0.0 | 4148.7 - 118.1 | 54 - 0 | 53 - 0 | 11,539.777 - 411.26 | 1.2 - -61.6 | 17.1 - -10.6 |

**Table S4**: Number of compounds with reported activity annotations against selected cell lines which resulted similar to **4b** and **2**, according to ECFP4-based similarity estimations.

| **Cell line** | **Intervals of activity** | **Number of compounds**  **similar to 4b**  **(ECFP4-fp)** | **Number of compounds similar to 2**  **(ECFP4-fp)** |
| --- | --- | --- | --- |
| HEK293 | < 1 µM | 56 | 56 |
|  | ≥1 µM - <10 µM | 93 | 93 |
|  | ≥10 µM - <50 µM | 311 | 311 |
|  | ≥ 50 µM | 251 | 251 |
| HeLa | < 1 µM | 561 | 561 |
|  | ≥1 µM - <10 µM | 1,170 | 1,170 |
|  | ≥10 µM - <50 µM | 1,335 | 1,335 |
|  | ≥ 50 µM | 1,080 | 1,080 |
| HepG2 | < 1 µM | 292 | 292 |
|  | ≥1 µM - <10 µM | 1,141 | 1,141 |
|  | ≥10 µM - <50 µM | 1,894 | 1,894 |
|  | ≥ 50 µM | 929 | 929 |
| SH-SY5Y | < 1 µM | 81 | 81 |
|  | ≥1 µM - <10 µM | 94 | 94 |
|  | ≥10 µM - <50 µM | 119 | 119 |
|  | ≥ 50 µM | 136 | 136 |
| THP-1 | < 1 µM | 91 | 91 |
|  | ≥1 µM - <10 µM | 146 | 146 |
|  | ≥10 µM - <50 µM | 146 | 146 |
|  | ≥ 50 µM | 151 | 151 |

**Table S5**: Percentages of compounds with reported activity annotations against selected cell lines, which resulted similar to **4b** and **2**, according to similarity estimations.

| **Cell line** | **Intervals of activity** | **Percentage of compounds similar to 4b (ECFP4-fp)** | **Percentage of compounds similar to 2 (ECFP4-fp)** | **Percentage of compounds to 4b**  **(TT-fp)** | **Percentage of compounds similar to 2**  **(TT-fp)** |
| --- | --- | --- | --- | --- | --- |
| HEK293 | < 1 µM | 7.9 | 7.9 | 12.9 | 0.0 |
|  | ≥1 µM - <10 µM | 13.1 | 13.1 | 12.9 | 0.0 |
|  | ≥10 µM - <50 µM | 43.7 | 43.7 | 29.0 | 33.3 |
|  | ≥ 50 µM | 35.3 | 35.3 | 45.2 | 66.7 |
| HeLa | < 1 µM | 13.5 | 13.5 | 8.3 | 8.4 |
|  | ≥1 µM - <10 µM | 28.2 | 28.2 | 21.6 | 16.3 |
|  | ≥10 µM - <50 µM | 32.2 | 32.2 | 43.5 | 32.8 |
|  | ≥ 50 µM | 26.0 | 26.0 | 26.6 | 42.5 |
| HepG2 | < 1 µM | 6.9 | 6.9 | 7.4 | 5.8 |
|  | ≥1 µM - <10 µM | 26.8 | 26.8 | 29.1 | 22.1 |
|  | ≥10 µM - <50 µM | 44.5 | 44.5 | 44.9 | 34.8 |
|  | ≥ 50 µM | 21.8 | 21.8 | 18.6 | 37.3 |
| SH-SY5Y | < 1 µM | 18.8 | 18.8 | 87.0 | 29.2 |
|  | ≥1 µM - <10 µM | 21.9 | 21.9 | 1.9 | 4.2 |
|  | ≥10 µM - <50 µM | 27.7 | 27.7 | 11.1 | 4.2 |
|  | ≥ 50 µM | 31.6 | 31.6 | 0.0 | 62.5 |
| THP-1 | < 1 µM | 17.0 | 17.0 | 16.7 | 12.5 |
|  | ≥1 µM - <10 µM | 27.3 | 27.3 | 33.3 | 12.5 |
|  | ≥10 µM - <50 µM | 27.3 | 27.3 | 10.0 | 6.3 |
|  | ≥ 50 µM | 28.3 | 28.3 | 40.0 | 68.8 |

**Figure S1: In vitro solubility of compounds 2 and 4b.** Compounds **2** and **4b** were diluted in PBS and solubility assessed by measuring optical density (OD) at 284 nm. Data are plotted as mean ± SD (n=3). Cpd, compound.

**
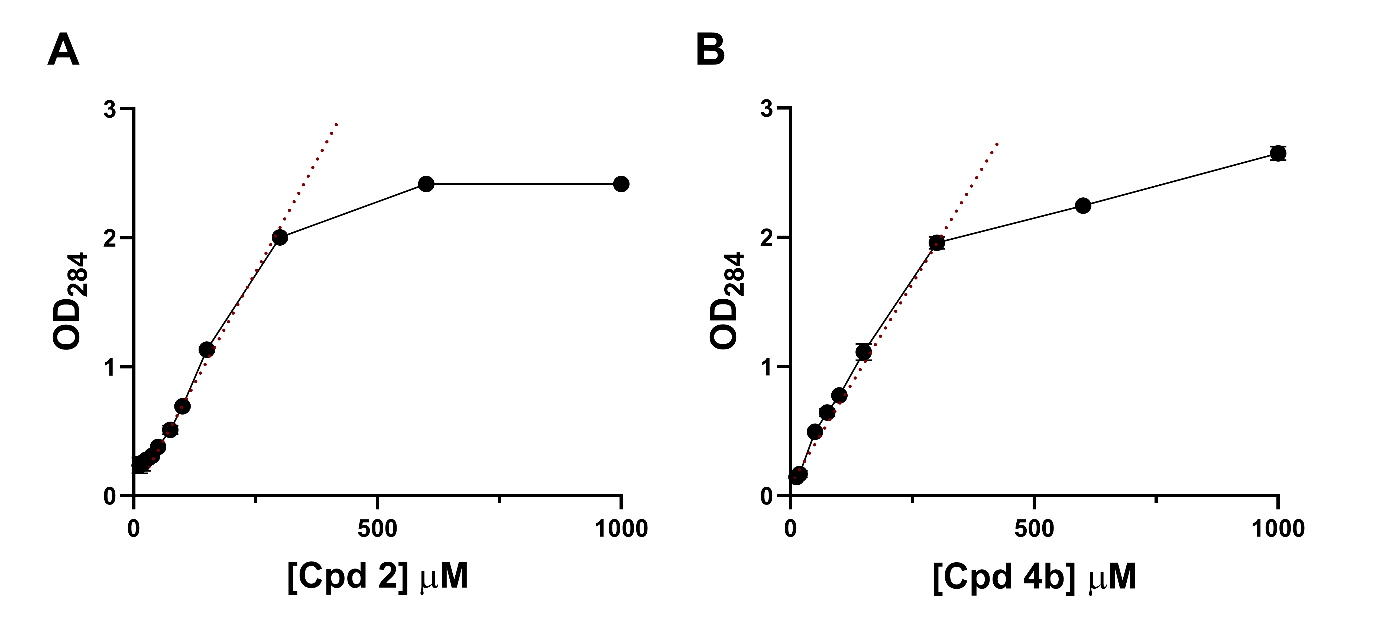
**

**Figure S2: Effect of compound 2 on OC cell viability.** A2780 OC cells over-expressing Rab25 were treated with compound **2** (10 and 50 µM) or DMSO for 24 h, fixed, stained with DRAQ5 and imaged with a Licor Odyssey Sa system. Images were quantified with Image Studio software. Nine technical repeats from n=3 independent experiments. p>0.05 by one-way ANOVA. Cpd, compound.

**
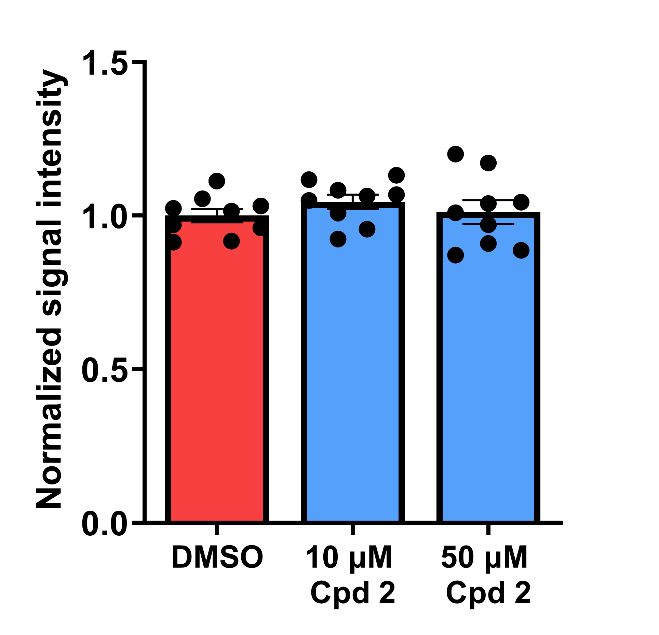
**

**Figure S3**: predicted binding pose of GM6001 (light grey sticks) and BB-94 (light orange sticks) into the ADAMTS5 active site.


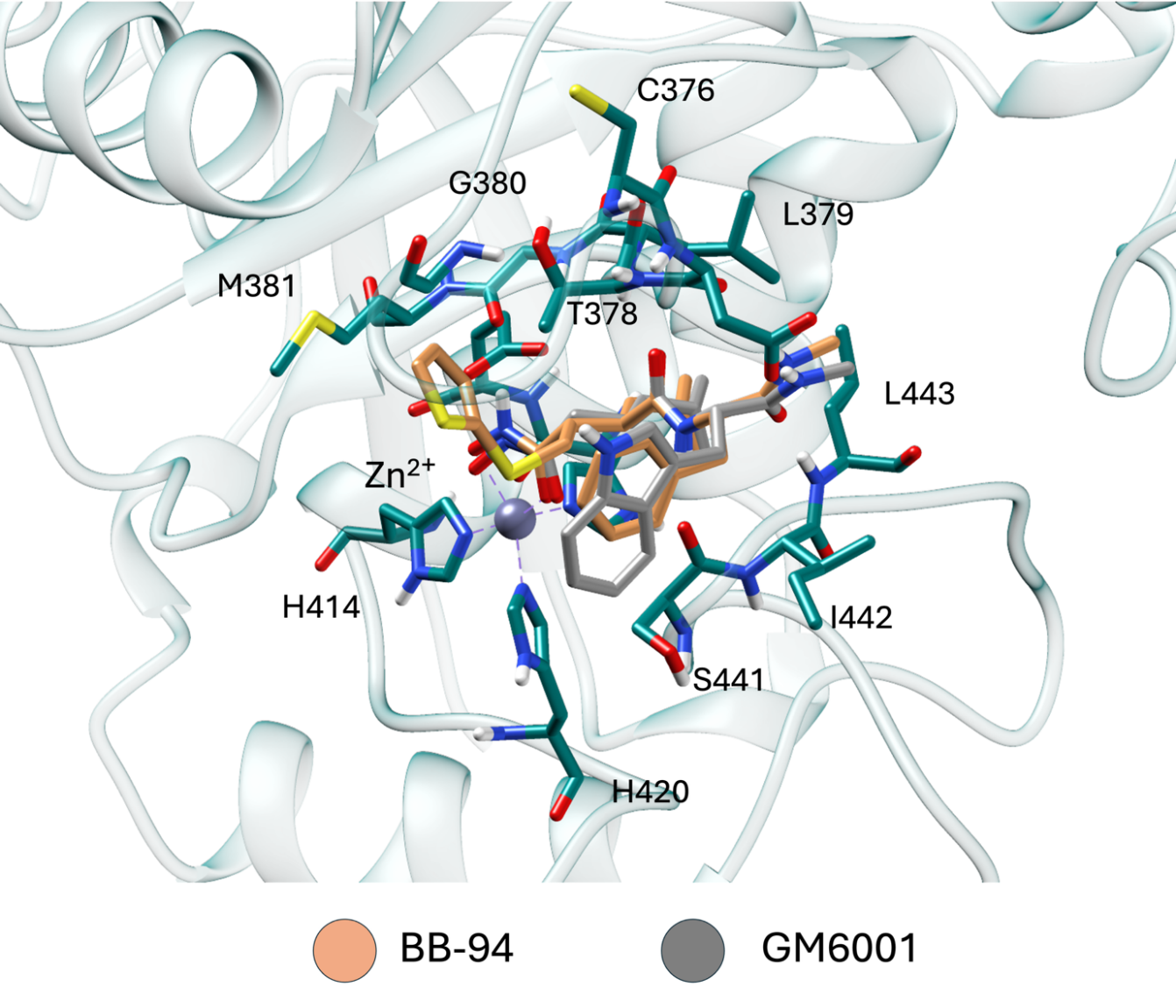


**Figure S4:** Full immunoblot corresponding to Figure 4D. Highlighted region (in red box) corresponds to the data reported in the main manuscript figure. Compounds were incubated with ADAMTS5 (1 nM) for 2 h at 37°C before addition of aggrecan (600 nM). Following SDS-PAGE and immunoblot, fragments were detected by the neoepitope aggrecan antibody BC3 that recognizes the new C-terminal fragment generated by ADAMTS5 cleavage at E^392^-A^393^.


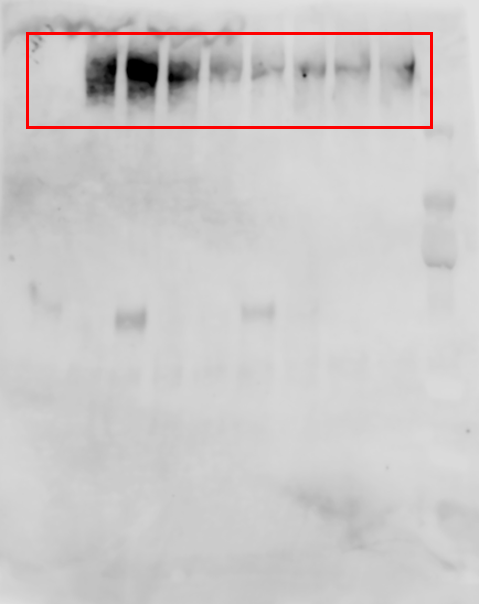


**Figure S5:** Full immunoblot corresponding to Figure 6A. Highlighted region (in red box) corresponds to the data reported in the main manuscript figure. Compounds were incubated with OA cartilage explants from patients undergoing knee surgery and the medium was analyzed by immunoblot with anti-AGEG neoepitope antibody (detecting aggrecan cleavage at E^1953^-A^1954^ in human aggrecan).


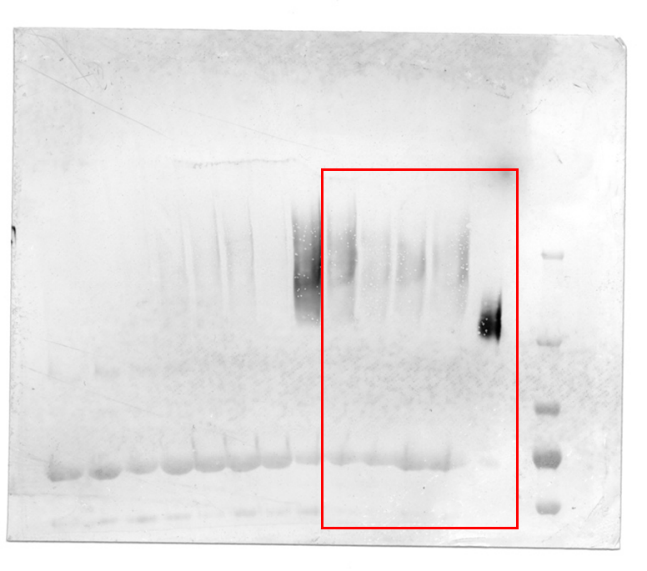


1. **NMR spectra of final compounds 1-4**

Compound **1**: ^1^H NMR (400 MHz, MeOD)

Compound **1**: ^13^C NMR (100 MHz, MeOD)

Compound **1**: ^1^H-^1^H COSY NMR (400 MHz, MeOD)

Compound **1**: ^1^H-^13^C HSQC NMR (MeOD)

Compound **2**: ^1^H NMR (400 MHz, MeOD)

Compound **2**: ^13^C NMR (100 MHz, MeOD)

Compound **2**: ^1^H-^1^H COSY NMR (400 MHz, MeOD)

Compound **3**: ^1^H NMR (400 MHz, MeOD)

Compound **3**: ^13^C NMR (100 MHz, MeOD)

Compound **3**: ^1^H-^1^H COSY NMR (400 MHz, MeOD)

Compound **3**: ^1^H-^13^C HSQC NMR (MeOD)

Compound **4**: ^1^H NMR (400 MHz, MeOD)

Compound **4**: ^13^C NMR (100 MHz, MeOD)

Compound **4**: DEPT 135 (100 MHz, MeOD)

Compound **4**: ^1^H-^1^H COSY NMR (400 MHz, MeOD)

Compound **4**: ^1^H-^13^C HSQC NMR (MeOD)

1. **HRMS spectra of final compounds**

HRMS spectrum of compound **1**

C_33_H_47_N_5_SO_8_

M_calc_ 673,31508

[M-H]^-^ 672,30726

HRMS spectrum of compound **2**

C_36_H_51_N_5_SO_13_

M_calc_ 793,31986

[M+H]^+^ 794,32768

[M+Na]^+^ 816,30963

HRMS spectrum of compound **3**

C_36_H_51_N_5_SO_13_

M_calc_ 793,31986

[M+H]^+^ 794,32768

[M+Na]^+^ 816,30963

[M+K]^+^ 832,28357

HRMS spectrum of compound **4**

C_42_H_61_N_5_O_18_S

M_calc_ 955.37268

[M+Na]^+^ 978.36245

C_42_H_61_N_5_O_18_S

M_calc_ 955.37268

[M+Na]^+^ 978.36245
